# Tonic endocannabinoid-mediated modulation of GABA release is independent of the CB_1_ content of axon terminals

**DOI:** 10.1038/ncomms7557

**Published:** 2015-04-20

**Authors:** Nora Lenkey, Tekla Kirizs, Noemi Holderith, Zoltán Máté, Gábor Szabó, E. Sylvester Vizi, Norbert Hájos, Zoltan Nusser

**Affiliations:** 1 Lendület Laboratory of Cellular Neurophysiology, Institute of Experimental Medicine, Hungarian Academy of Sciences, Budapest H1083, Hungary; 2 János Szentágothai School of Neurosciences, Semmelweis University, Budapest H1085, Hungary; 3 Division of Medical Gene Technology, Institute of Experimental Medicine, Hungarian Academy of Sciences, Budapest H1083, Hungary; 4 Laboratory of Drug Research, Institute of Experimental Medicine, Hungarian Academy of Sciences, Budapest H1083, Hungary; 5 Lendület Laboratory of Network Neurophysiology, Institute of Experimental Medicine, Hungarian Academy of Sciences, Szigony street 43, Budapest H1083, Hungary

## Abstract

The release of GABA from cholecystokinin-containing interneurons is modulated by type-1 cannabinoid receptors (CB_1_). Here we tested the hypothesis that the strength of CB_1_-mediated modulation of GABA release is related to the CB_1_ content of axon terminals. Basket cell boutons have on average 78% higher CB_1_ content than those of dendritic-layer-innervating (DLI) cells, a consequence of larger bouton surface and higher CB_1_ density. The CB_1_ antagonist AM251 caused a 54% increase in action potential-evoked [Ca^2+^] in boutons of basket cells, but not in DLI cells. However, the effect of AM251 did not correlate with CB_1_ immunoreactivity of individual boutons. Moreover, a CB_1_ agonist decreased [Ca^2+^] in a cell type- and CB_1_-content-independent manner. Replica immunogold labelling demonstrated the colocalization of CB_1_ with the Cav2.2 Ca^2+^ channel subunit. Our data suggest that only a subpopulation of CB_1_s, within nanometre distances from their target Cav2.2 channels, are responsible for endocannabinoid-mediated modulation of GABA release.

## Abstract (web summary)

A simple model for receptor number–function relationships dictates a positive correlation between receptor content and ligand effect. Here, the authors demonstrate that the degree of type-1 cannabinoid receptor (CB_1_)-modulated GABA release cannot be predicted from CB^1^ content of axon terminals.

## Graphical abstract

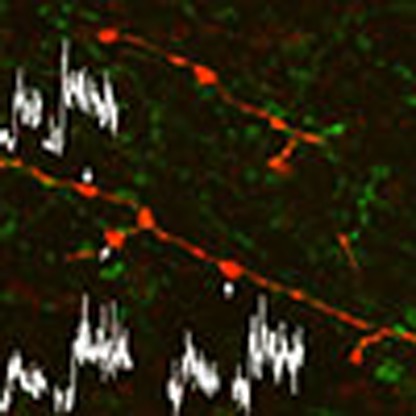

The effect of a neuroactive substance on its target cells depends on its spatio-temporal concentration profile, its affinity to the receptors and the number of receptors on the target cells. According to this simple view, the more receptors a cell expresses on its surface, the larger the effect of the ligand. When this simple model was tested for ion-channel-forming receptors, it was found that the number of synaptic AMPA receptors strongly predicts the size of the postsynaptic AMPA receptor-mediated responses^1^,^2^,^3^,^4^,^5^,^6^ and that more synaptic GABA_A_ receptors confer larger postsynaptic inhibitory responses^7^,^8^. The consequence of the activation of ionotropic receptors can be relatively easily monitored by patch-clamp electrophysiological or imaging techniques, allowing the determination of the ‘number–function’ relationships for these receptors. In contrast to ion-channel-forming receptors, it is much more challenging to quantify the effects of metabotropic receptor activation, since they act through second messenger pathways, which could contain signal amplification/saturation steps. Thus, it is much more difficult to determine their ‘number–function’ relationships, requiring the quantitative measurement of their function as well as their numbers in small, functionally relevant subcellular compartments.

G-protein-coupled receptors (GPCRs) form a diverse family, with hundreds of genes expressed in the central nervous system^9^. A significant fraction of GPCRs are located on presynaptic axon terminals, where they effectively control neurotransmitter release^10^,^11^,^12^,^13^,^14^. The mechanisms of regulation of release have been extensively studied, revealing numerous mechanisms, including the direct or indirect modulation of presynaptic voltage-gated Ca^2+^ or K^+^ channels, or the modification of the release machinery through, for example, phosphorylation^11^,^15^,^16^,^17^. To investigate the ‘number–function’ relationship for a given GPCR, the mechanisms of action need to be quantitatively understood.

In a recent study^18^, we examined the mechanisms of action of presynaptic type-1 cannabinoid receptors (CB_1_) on cholecystokinin (CCK)-expressing GABAergic interneurons (INs) of the CA3 region of the hippocampus. Our results revealed that tonic CB_1_ activation results in a reduction of Ca^2+^ influx through N-type Ca^2+^ channels (Cav2.2 subunits), which is primarily responsible for the reduction of GABA release. The change in Ca^2+^ influx can be measured with two-photon microscopy at the level of individual axon terminals in acute slices. It is well known that CB_1_ is strongly expressed by a diverse population of hippocampal CCK-positive (CCK+) INs in their axons^19^, resulting in a sparse axonal labelling of the hippocampal neuropil. This sparse labelling provides a unique opportunity for *post hoc* measurements of the CB_1_ content of *in vitro* imaged boutons. In addition, CB_1_ seemed to be a good candidate for such a receptor ‘number–function’ study, since substantial heterogeneity has been reported in the degree of CB_1_ modulation of GABA release from distinct CCK+ IN subtypes^20^,^21^,^22^, similar to that of glutamatergic boutons of the cerebellum^23^. In addition, the CB_1_ content of individual boutons also seems to be variable^24^,^25^.

In this study, we quantified the CB_1_ content of axon terminals and found remarkable heterogeneity among individual boutons of a single cell, individual cells of a single cell type and different cell types. In addition, the amplitude of action potential (AP)-evoked [Ca^2+^] and its modulation by CB_1_ also exhibits substantial variability within- and between cell types. Basket cell axon terminals contain more CB_1_, have smaller [Ca^2+^] transients and respond more strongly to CB_1_ antagonism compared with dendritic-layer-innervating (DLI) cells. Despite this, the degree of CB_1_ modulation does not depend on the total amount of CB_1_ of axon terminals. We also demonstrate the colocalization of CB_1_ with Cav2.2 Ca^2+^ channel subunit clusters in a confined area of the presynaptic terminal membranes; this area is likely to be the presynaptic active zone (AZ).

## Results

### Variability in CB_1_ content of CCK+ GABAergic boutons

**Figure 1:**
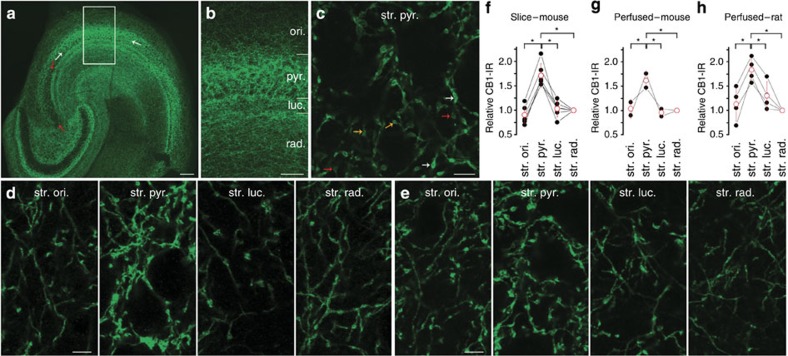
Variability in CB _1_ content of axon terminals in the CA3 region of the hippocampus. (**a**) CB_1_ immunofluorescent labelling is different in distinct layers of the mouse hippocampus, and it is stronger in the distal CA3 (between white arrows) than in the proximal CA3 (between red arrows). In the distal CA3, the stratum pyramidale is more intensely labelled than the strata oriens, lucidum and radiatum. (**b**) The boxed area in **a** is shown at a higher magnification. (**c**) There is high variability in the CB_1_ content among individual boutons. Axon terminals with weak (red arrows), strong (white arrows) and intermediate (orange arrows) CB_1_ immunoreactivity (CB_1_-IR) can be found in the stratum pyramidale of the distal CA3. (**d**,**e**) CB_1_ immunofluorescent reactions on sections obtained from a transcardially perfused mouse (**d**) and a rat (**e**). Individual boutons in stratum pyramidale have strong CB_1_-IR, whereas axon terminals in strata oriens, lucidum and radiatum contain comparable levels of CB_1_-IR. Note that within each region, there is bouton-to-bouton variability in the CB_1_ content. (**f**–**h**) Relative CB_1_-IR of individual boutons in different layers of the distal CA3 area of the hippocampus normalized to the mean CB_1_ intensity of boutons in stratum radiatum. The CB_1_ content similarly changes between layers in slices derived from *in vitro* imaging experiments (**f**), perfused mouse (**g**) and rat (**h**) sections. Randomized block design ANOVA was performed on the data sets before normalization, and significant difference between groups was found for the *in vitro* mouse (*P*<0.001, *n*=6), perfused mice (*P*<0.001, *n*=5) and perfused rat (*P*<0.001, *n*=4) data. Tukey *post hoc* test revealed a significant difference between stratum pyramidale and stratum oriens (*P*<0.001, *P*<0.001 and *P*=0.001), pyramidale and lucidum (*P*<0.001, *P*<0.001 and *P*=0.005) and pyramidale and radiatum (*P*<0.001, *P*<0.001 and *P*<0.001) for the *in vitro* slices, perfused mice and perfused rat sections, respectively. str. ori., stratum oriens; str. pyr., stratum pyramidale; str. luc, stratum lucidum; str. rad., stratum radiatum. Error bars in **f**–**h** represent s.d. Scale bars, 100 μm (**a**), 50 μm (**b**), 5 μm (**c**–**e**). *denotes *P*<0.05.

Although previous studies have suggested variability in the CB_1_ content of GABAergic axons in the CA1 region of the rat hippocampus^24^,^25^, we aimed at providing a systematic, quantitative analysis of the CB_1_ content of axon terminals in distinct layers in the CA3 area (Fig. 1). In low-magnification light microscopic (LM) images of hippocampal sections immunolabelled for CB_1_, the stratum pyramidale in the distal CA3 (closer to the CA2 area) appeared more intensely labelled in many slices than either the dendritic layers or the stratum pyramidale of the proximal CA3 (closer to the dentate gyrus; Fig. 1a). To reduce variability in CB_1_ immunoreactivity (CB_1_-IR) originating from differences between distal and proximal CA3 regions, we confined our analysis to the distal part of the CA3 area. Since the appearance of immunofluorescent signal at low magnifications is the consequence of both the density of boutons and the CB_1_-IR of individual boutons, the observed differences in signal intensities between strata oriens, pyramidale, lucidum and radiatum do not necessarily reflect differences in the CB_1_ content of CCK+ axon terminals. To characterize the CB_1_-IR of individual boutons, we obtained high-resolution confocal Z-image stacks and measured the integral of fluorescence (see Methods) of randomly selected boutons in these four strata in immersion-fixed mouse slices following *in vitro* imaging experiments (Fig. 1a–c,f), and in sections from transcardially perfused mice (Fig. 1d,g) and rats (Fig. 1e,h). The mean CB_1_-IR of individual boutons was significantly higher in stratum pyramidale compared with strata oriens and radiatum in the distal CA3 area (randomized block analysis of variance (ANOVA): *P*<0.001, Tukey *post hoc* test: *P*<0.001).

**Figure 2:**
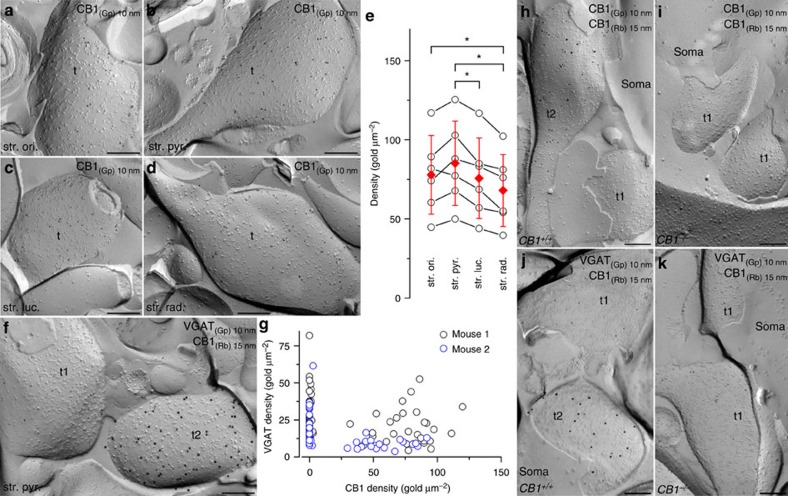
High-resolution immunogold localization of CB _1_ in the distal CA3 area of the mouse hippocampus. (**a**–**d**) Variability in the CB_1_ content of boutons in distinct layers of the distal CA3 area. The CB_1_ labelling on the P-face of axon terminals (t) in stratum pyramidale (**b**) is more intense than in strata oriens (**a**), lucidum (**c**) and radiatum (**d**). (**e**) Randomized block design ANOVA followed by Tukey *post hoc* test (*n*=6) showed significant difference between strata pyramidale and lucidum (*P*=0.019), between strata pyramidale and radiatum (*P*<0.001) and between strata oriens and radiatum (*P*=0.014). The labelling in all four layers was significantly higher than the background (ANOVA: *P*<0.001; Tukey’s *post hoc* test: *P*<0.001; *n*=6 mice; 1.0±0.3 gold per μm^2^). Red diamonds indicate the mean±s.d. (**f**) Double-labelling experiments for CB_1_ (15 nm gold) and vesicular GABA transporter (VGAT, 10 nm gold) reveal that the CB_1_-immunolabelled profiles are GABAergic axon terminals (t2), but not all VGAT-labelled terminals contain CB_1_ (t1). (**g**) Background-subtracted density values for CB_1_ and VGAT of VGAT-immunopositive axon terminals are shown from two animals. Symbols represent individual boutons. (**h**–**k**) The CB_1_ immunolabelling is abolished in *CB*_*1*_^*−/−*^ mice. A perisomatic axon terminal is co-labelled with two CB_1_ antibodies in a *CB*_*1*_^*+/+*^ mouse (t2 in **h**), while another is unlabelled (presumed parvalbumin-positive axon terminals, t1 in **h**). In *CB*_*1*_^*−/−*^ mice only non-labelled profiles could be observed in the stratum pyramidale (t1 in **i**). A VGAT (10 nm gold) immunolabelled axon terminal is strongly CB_1_ immunopositive (15 nm gold, t2 in **j**) in a *CB*_*1*_^*+/+*^ mouse, but all VGAT-positive boutons are immunonegative in the *CB*_*1*_^*−/−*^ tissue (t1 in **k**). str. ori., stratum oriens; str. pyr., stratum pyramidale; str. luc, stratum lucidum; str. rad., stratum radiatum; Rb, rabbit; Gp, guinea pig. All scale bars, 200 nm. *denotes *P*<0.05.

**Figure 3:**
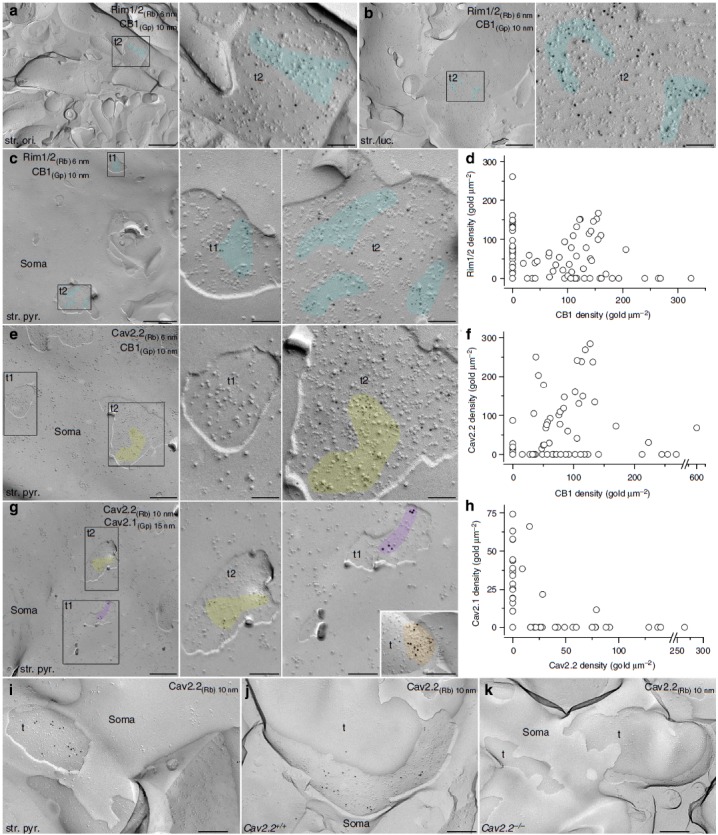
SDS-FRL localization of RIM1/2 and Cav2.2 subunit on CB _1_ -immunopositive axon terminals in the distal CA3 area of the mouse hippocampus. (**a**–**b**) Immunogold particles labelling CB_1_ (10 nm gold, t2) are present in the active zones (AZ; blue, labelled by Rim1/2 with 6 nm gold) of axon terminals. (**c**) P-face sections of CB_1_-immunopositive (t2 in **c**) and negative (t1 in **c**) axon terminals are attached to an E-face somatic plasma membrane. Both axon terminals contain a Rim1/2 positive AZ (blue). (**d**) Background-subtracted density values for CB_1_ and Rim1/2 measured in P-face axon terminal sections attached to the E-face membrane of pyramidal cell somata. A total of 69.2% of axon terminal fragments were double negative, while 9.2% were only Rim1/2, 7.2% were only CB_1_-positive and the remaining 14.4% contained both Rim1/2 and CB_1_ labelling. (**e**) A P-face axon terminal fragment attached to an E-face somatic membrane is co-labelled for CB_1_ and Cav2.2 subunit (t2), while another is unlabelled (t1). (**f**) Background-subtracted densities for CB_1_ and Cav2.2 subunit within P-face axon terminal sections. The CB_1_-positive profiles were either Cav2.2 positive or negative, but the majority (84.2%) of the Cav2.2 subunit-immunopositive profiles were CB_1_ positive. (**g**) Double-labelling experiments reveal that the P-face axon terminal fragments attached to E-face somatic membranes are labelled for either Cav2.2 (t2) or Cav2.1 (t1) subunit. The AZ of an excitatory axon terminal (t) contains large number of gold particles for both Cav2.2 and Cav2.1 subunits (orange, inset). (**h**) Background-subtracted density values for Cav2.2 and Cav2.1 of P-face bouton fragments. (**i**–**k**) The Cav2.2 subunit immunolabelling is abolished in *Cav2.2*^*−/−*^ mice. Perisomatic axon terminals are labelled for Cav2.2 in *CCK-BAC/DsRedT3* (t in **i**) and *Cav2.2*^*+/+*^ mice (t in **j**). In *Cav2.2*^*−/−*^ mice only non-labelled profiles were observed in stratum pyramidale. Enlarged views of the boxed areas in **a**–**c**, **e** and **g** are shown on the right. str. ori., stratum oriens; str. luc, stratum lucidum; str. pyr., stratum pyramidale; Rb, rabbit; Gp, guinea pig. Scale bars, 500 nm (**a**–**c**,**e**,**g**); 100 nm, enlarged views of **a**–**c** and **e**; 200 nm, **i**–**k** and enlarged views and inset in **g**.

Our fluorescent measurements provide an estimate of the total CB_1_ content of the axon terminals, which is a function of the CB_1_ surface density, the surface area of the bouton and potential intracellular pool of receptors. Thus, the higher CB_1_-IR in the stratum pyramidale (Fig. 1) could be the consequence of an elevated CB_1_ density and/or larger boutons and/or more intracellular receptors. To directly test the CB_1_ density in membranes of axon terminals, we have carried out electron microscopic (EM) freeze-fracture replica immunogold labelling (SDS-FRL) for CB_1_ in four distinct layers of the distal CA3 area in mice (Fig. 2) and rats (Supplementary Fig. 1). Gold particles for CB_1_ were enriched in some protoplasmic-face (P-face) membrane segments, consistent with the cytoplasmic location of the epitope recognized by the antibodies. Gold particles were apparently randomly distributed over these CB_1_-positive fractured membranes, without large areas of elevated or reduced densities (Fig. 2a–d,f; Supplementary Fig. 1a–d,f). The strongly CB_1_-positive P-face membranes are GABAergic, because the CB_1_-positive profiles were also immunopositive for the vesicular inhibitory amino-acid transporter (VIAAT or vesicular GABA transporter; Fig. 2f,g,j; Supplementary Fig. 1f,g). Furthermore, the CB_1_-positive profiles assumed to be axon terminals of CCK+ INs for the following reasons: (1) the size of the fractured membranes is consistent with them being axons, axon terminals, dendritic spines or small dendrites, but inconsistent with somata, large or medium diameter dendrites; (2) the frequency of finding a CB_1_-positive profile is inconsistent with the labelling of excitatory terminals; (3) CB_1_-positive membranes are often attached to the somatic exoplasmic-face (E-face) membranes (Fig. 3c,e; Supplementary Figs 2e and 3a,d), a feature that never occurs for postsynaptic profiles. We quantified the densities of CB_1_ immunogold particles on P-face structures in all four layers of the distal CA3 in mice (Fig. 2e) and rats (Supplementary Fig. 1e) and found that they were significantly higher than the background labelling (one-way ANOVA: *P*<0.001; Tukey’s *post hoc* test: *P*<0.01) determined on surrounding E-face profiles. Furthermore, the boutons in stratum pyramidale contained significantly higher gold particle densities than those in strata lucidum and radiatum (randomized block ANOVA, *P*<0.05; Tukey’s *post hoc* test, *P*<0.05). The specificity of the immunolabelling for two antibodies raised against the CB_1_ was confirmed using *CB*_*1*_^*−/−*^ and *CB*_*1*_^*+/+*^ mice (see Methods and Fig. 2h–k). Double labelling for CB_1_ and the presynaptic AZ marker Rim1/2 revealed an extensive colocalization (Fig. 3a–d; Supplementary Fig. 2), providing evidence for the presence of CB_1_ in presynaptic AZs. Although it has been demonstrated that the influx of Ca^2+^ into CCK+ axon terminals is mediated by N-type Ca^2+^ channels^18^, the exact location of the pore forming Cav2.2 subunit and its relative position to CB_1_ is unknown. Therefore, we performed colocalization of CB_1_ and Cav2.2 and found that many CB_1_-positive terminals also contain Cav2.2 (Fig. 3e; Supplementary Figs 3c,d and 5). The fact that not every CB_1_-positive profile contains Cav2.2 (Fig. 3f; Supplementary Fig. 3e) is likely to be the consequence of the lack of AZs in a large fraction (70–80%) of randomly fractured axon terminal membranes. The Cav2.2-positive profiles, however, were almost always CB_1_ positive. The segregation of the Cav2.1 and Cav2.2 subunits in axon terminal membrane segments fractured onto pyramidal cell (PC) somata (Fig. 3g,h; Supplementary Fig. 3f,g,i) provide morphological evidence for the exclusive role of these two subunits in parvalbumin and CCK+ basket cell axon terminals, respectively. The specificity of the immunolabelling for Ca^2+^ channel subunits was confirmed using knockout animals (for Cav2.2 see Methods and Fig. 3i–k; for Cav2.1; see ref. 26).

Having established a significant difference in the CB_1_ density of axon terminals in stratum pyramidale and dendritic layers, we also investigated whether additional differences in the size of the boutons exist. We performed three-dimensional (3D) EM reconstructions of CCK+ boutons from serial ultrathin sections in the distal CA3 region of the hippocampus (*n*=22 and 21 boutons in strata pyramidale and radiatum, respectively, *n*=3 mice; Supplementary Fig. 4). Boutons were usually flat (especially in stratum pyramidale) and had variable shapes (Supplementary Fig. 4a,b). Our quantification revealed that the surface area of boutons, the bouton volume, the total AZ area and the number of AZs per boutons were all significantly larger, whereas the total AZ area—bouton volume ratio and the total AZ area—bouton surface ratio were smaller in terminals in stratum pyramidale compared with those in stratum radiatum (Supplementary Fig. 4c–h; unpaired *t*-test, *P*≤0.01). Our 3D reconstructions revealed that most CCK+ boutons in the stratum pyramidale contained multiple AZs, which had variable shapes and sizes. Consistent with this, the shapes and sizes of Rim1/2-immunolabelled AZs (Fig. 3a–c) and Cav2.2-enriched areas (Fig. 3e; Supplementary Figs 3c–d,f–g and 5) in our replicas also showed great variability, and many perisomatic boutons had multiple AZs.

Our SDS-FRL experiments and EM 3D reconstructions extend our LM immunofluorescent results by showing that CB_1_ receptors are located in the plasma membranes of axon terminals, and that density and bouton surface differences primarily determine the observed differences in the total CB_1_-IR of boutons.

### Basket cell boutons contain more CB_1_ than those of DLI cells

GABAergic INs expressing CCK and CB_1_ consist of distinct subtypes that innervate distinct subcellular domains of postsynaptic PCs and arborize mainly in distinct layers; basket, DLI, perforant-path-associated and mossy fiber-associated cells have been described^20^,^27^,^28^,^29^,^30^. In the present study, we focus on basket and DLI cells, which mainly innervate the perisomatic and dendritic region of PCs, respectively. The stronger CB_1_ labelling in stratum pyramidale (Fig. 1) suggests that axon terminals of basket cells contain more CB_1_ than those of DLI cells. Although the axons of these cell types mainly arborize in distinct layers, a remarkable amount of axon terminals can be found in other layers^29^,^31^. For example, basket cells also have axons in strata lucidum, proximal radiatum and oriens, whereas DLI cells also arborize in stratum pyramidale. Thus, the observed layer-specific differences cannot be directly used to infer cell type-specific differences. Furthermore, within each hippocampal layer, we detected large variability within the labelling intensity among boutons; the mean coefficient of variations (CVs) were 39%, 50%, 49% and 49% in strata pyramidale, oriens, lucidum and radiatum, respectively. The origin of this variability cannot be determined without examining individual identified cells.

**Figure 4:**
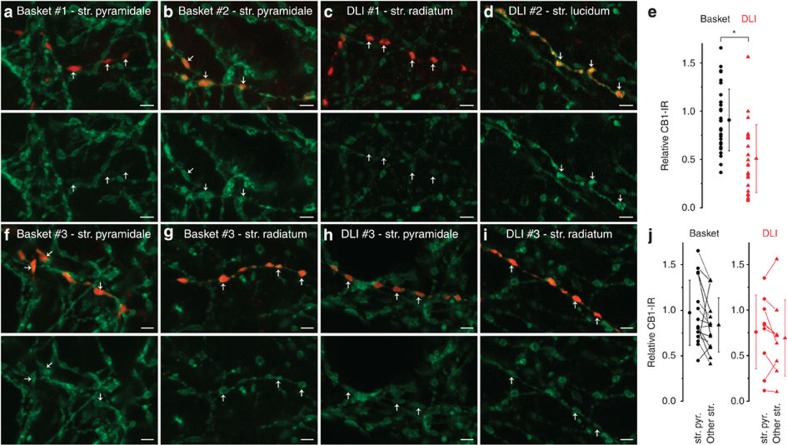
The CB _1_ content of axon terminals in the distal CA3 is cell type-dependent, but independent of hippocampal layers. (**a**–**d**) Variability in the CB_1_ content of axon terminals of different cells. *Post hoc* immunohistochemical visualization of CB_1_ (green) in boutons (red) of *in vitro* [Ca^2+^] imaged and biocytin-filled GABAergic interneurons. Weakly (**a**,**c**) and strongly (**b**,**d**) CB_1_-immunopositive basket (**a**,**b**) and dendritic-layer-innervating (DLI, **c**,**d**) cells are shown. Lower panels display only the CB_1_ immunoreactivity (CB_1_-IR) for better visualization of the differences in the CB_1_ content. Arrows in upper and lower panels indicate the same boutons. (**e**) Basket cell axon terminals are stronger for CB_1_ than those of DLI cells in the distal CA3. The CB_1_-IR of biocytin-loaded boutons was normalized to the mean CB_1_ intensity of randomly selected non-biocytin-filled boutons in stratum pyramidale within the same section. (**f**–**i**) The strength of CB_1_-IR of boutons of a given cell is similar in different hippocampal layers. Both basket and DLI cells had axons in strata pyramidale (**f**,**h**) and radiatum (**g**,**i**). Lower panels show only the CB_1_-IR for better visualization of the differences in the CB_1_ content. (**j**) CB_1_-IR of axon terminals of individual cells is not significantly different in stratum pyramidale versus dendritic layers in basket and DLI cells. Error bars in **e** and **j** represent s.d. str. pyr., stratum pyramidale. Other str.: strata oriens, lucidum and radiatum. All scale bars, 2 μm. *denotes *P*<0.001.

To unequivocally identify the CB_1_ content of axon terminals of distinct cell types, CCK+ INs were recorded with a biocytin- and Fluo-5F-containing intracellular solution. Following *in vitro* electrophysiological characterization and two-photon [Ca^2+^] measurements, we immersion-fixed the slices and immunolabelled them for CB_1_ (Fig. 4). Qualitatively, cells with weakly and strongly immunoreactive boutons were found in both IN subtypes (Fig. 4a–d). When cells were analysed quantitatively in the layers where their main axonal arbors were (basket: str. pyramidale; DLI: str. radiatum), large heterogeneity was observed in both basket and DLI cell populations, with CVs of 35% and 69%, respectively. The mean CB_1_-IR of basket cell axon terminals was 91±32% of the average reactivity of the non-biocytin-filled boutons in the stratum pyramidale (for normalization see Methods), whereas it was only 51±35% for DLI cells (Fig. 4e). These results demonstrate that axon terminals of basket cells are more strongly CB_1_ immunoreactive than those of DLI cells (unpaired *t*-test, *P*<0.001, *n*_basket_=32, *n*_DLI_=25), when measured in the layer where they mainly arborize. However, as mentioned above, both cell types also possess axons outside their main layers, the CB_1_-IR of which was the subject of our next measurements. We selected bouton strings distant from the main arbor and compared their reactivity with those that were in stratum pyramidale for basket (Fig. 4f,g) and stratum radiatum for DLI cells (Fig. 4h,i) and found no significant difference in the average CB_1_-IR between different layers (Fig. 4j; paired *t*-test, basket: *P*=0.11, *n*=15; DLI: *P*=0.31, *n*=9).

### Intrabouton [Ca^2+^] is smaller in basket than in DLI cells

**Figure 5:**
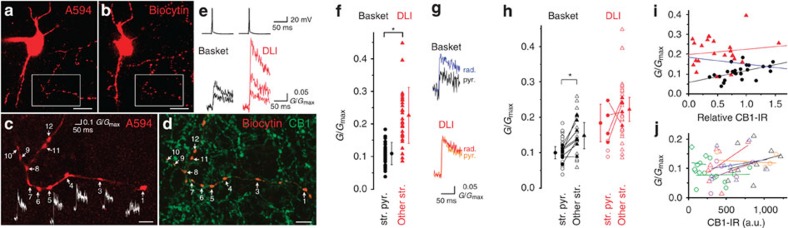
Action potential-evoked [Ca ^2+^ ] transients are smaller in axon terminals of basket compared with DLI cells. (**a**) Two-photon Z-stack maximum intensity projection image of a basket cell loaded with Alexa Fluor 594. (**b**) Confocal Z-stack maximum intensity projection image of the two-photon-imaged cell after fixation and visualization of the biocytin. (**c**) The imaged boutons from **a** are shown at a higher magnification with the corresponding [Ca^2+^] transients. The presented Ca^2+^ traces are the averages of two individual traces for each bouton. Note the variability in the peak transients within a bouton string. (**d**) *Post hoc* immunohistochemical visualization of CB_1_ (green) in the imaged and biocytin-filled (red) boutons. (**e**) Variability in action potential-evoked [Ca^2+^] transients in axon terminals of basket (imaged in stratum pyramidale) and DLI (imaged in strata radiatum or lucidum) cells. Every trace represents a single cell (averages of 18–30 individual traces). (**f**) [Ca^2+^] transients of DLI cells are significantly larger compared with basket cells. (**g**) Mean [Ca^2+^] transients measured in axon terminals in strata pyramidale and radiatum in a basket and a DLI cell. (**h**) Peak [Ca^2+^] transients in basket cell boutons are smaller in stratum pyramidale compared with the dendritic layers. Open symbols indicate independent measurements (individual cells measured in either layer), while filled symbols represent cells, in which boutons in both strata were measured (basket: *n*=8; DLI: *n*=4). * indicates significant difference (paired *t*-test, *P*<0.01, *n*=8) between strata pyramidale and dendritic layers for basket cells. (**i**) The amplitude of [Ca^2+^] transients is independent of the CB_1_ immunoreactivity of the cells when all cells are analysed together. When basket and DLI cells are analysed separately, significant positive correlation (Spearman correlation *ρ*=0.55, *P*=0.006) was found in baskets, but not in DLI cells. (**j**) No significant correlation was found between *G*/*G*_max_ and CB_1_ content at the level of individual boutons in all analysed basket cells. Each cell is colour coded and open symbols represent individual boutons. Error bars in **f** and **h** represent s.d. A594, Alexa Fluor 594; str. pyr., stratum pyramidale. Other str.: strata oriens, lucidum and radiatum. Scale bars, 20 μm (**a**,**b**); 5 μm (**c**,**d**).

Our initial step towards assessing the function of presynaptic CB_1_ was to measure AP-evoked, volume-averaged [Ca^2+^] transients in individual CCK+ and CB_1_-immunoreactive boutons in acute slices obtained from *BAC-CCK-DsRed* transgenic mice^18^,^32^. Calcium imaging experiments were performed with a two-photon laser scanning microscope. Cells were intracellularly loaded with the calcium-sensitive dye Fluo-5F (100 μM) and Alexa-594 (25 μM, Fig. 5a) for an initial 1 h equilibration period, then bouton strings were selected and imaged in strata pyramidale, radiatum, lucidum or oriens (Fig. 5c). Each AP reliably evoked [Ca^2+^] transients in every bouton of the string, the amplitude of which showed bouton-to-bouton variability (basket: CV=0.37±0.13, DLI: CV=0.32±0.12). The peak [Ca^2+^] transients also showed cell-to-cell variability within a cell type (basket: CV=0.31, DLI: CV=0.38; Fig. 5e,f). DLI cells had, on average 107% larger peak [Ca^2+^] transients compared with basket cells (Fig. 5f; unpaired *t*-test, *P*<0.001, *n*_basket_=40, *n*_DLI_=31). In these experiments, basket cell boutons were measured in stratum pyramidale, whereas those of DLI cells were recorded in the dendritic layers. We also measured the [Ca^2+^] in layers where the cells had only a small fraction of their axons and found significantly (paired *t*-test, *P*=0.004, *n*=8) higher [Ca^2+^] for basket cells in the dendritic layers (Fig. 5g,h). The [Ca^2+^] was also somewhat larger for DLI cells in the dendritic layers (str. pyramidale *G*/*G*_max_=0.184±0.053; dendritic layers *G*/*G*_max_=0.222±0.034, *n*=4), but this did not reach statistical significance (Fig. 5g,h).

After the imaging experiments, the slices were immersion fixed and biocytin was visualized for *post hoc* determination of the cell type based on the axonal arbor (Fig. 5b). The sections were also immunolabelled for CB_1_ to assess the CB_1_-IR of the biocytin-filled boutons of the imaged cells (Fig. 5d). Analysis of the peak [Ca^2+^] and CB_1_-IR revealed no significant correlation when both cell types were analysed together (Fig. 5i, blue regression line, Spearman’s correlation, *ρ*=−0.18, *P*=0.23, *n*=47). When the correlation analysis was performed on the two IN subtypes separately, there was significant positive correlation between peak [Ca^2+^] and CB_1_-IR in the basket cell group (*ρ*=0.55, *P*=0.006, *n*=24), but not for the DLI cells (*ρ*=−0.017, *P*=0.88, *n*=23). Analysis at the individual bouton level showed no correlation in all analysed basket cells (Fig. 5j; *ρ*-values were:−0.02, 0.39, 0.30, −0.26, 0.09, 0.46 and 0.21).

### Cell type-dependent, CB_1_ content-independent effect of AM251

**Figure 6:**
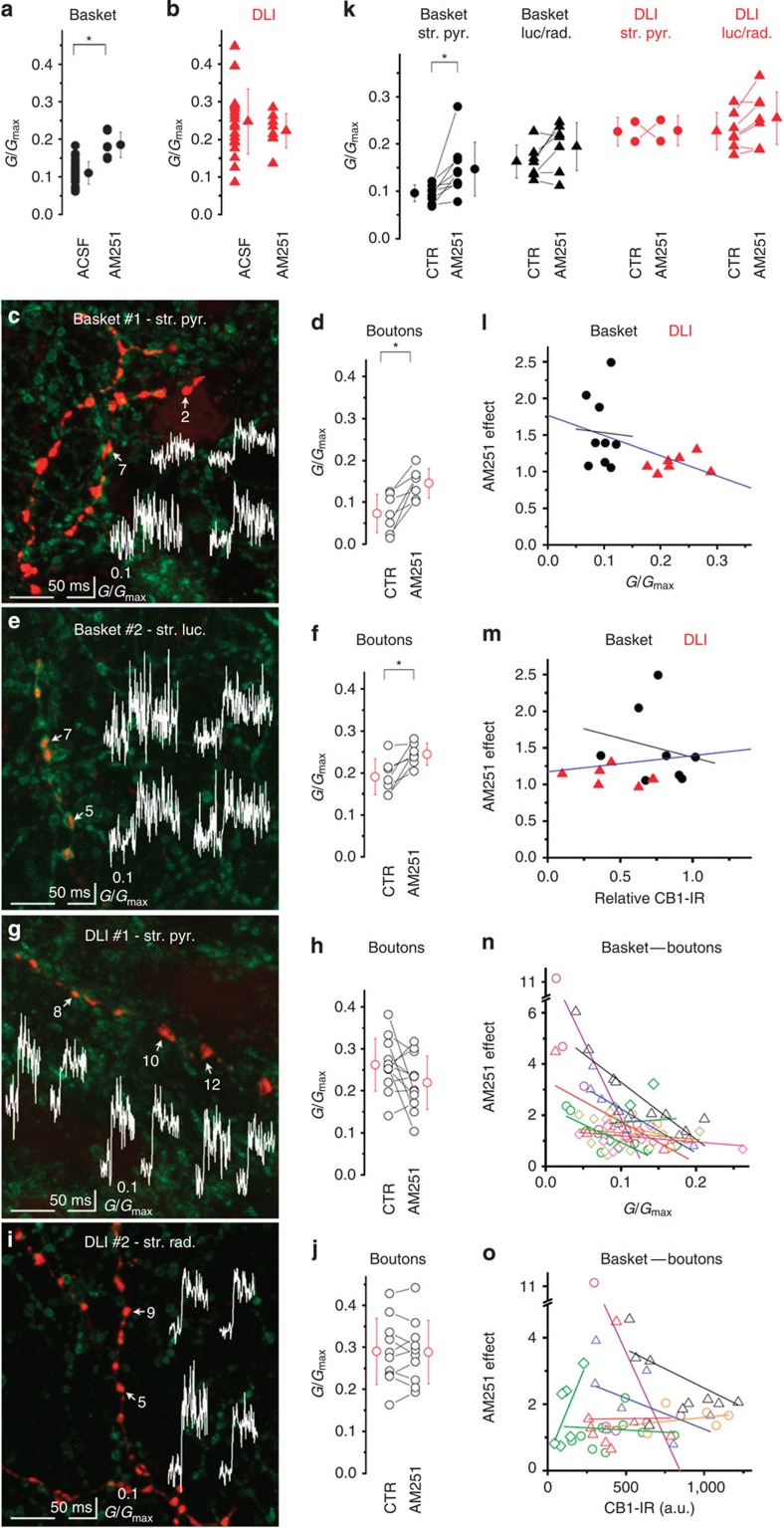
The CB _1_ antagonist AM251 significantly increases [Ca ^2+^ ] transients in axon terminals of basket, but not those of DLI cells in the CA3 area. (**a**,**b**) The amplitude of [Ca^2+^] transients is larger in basket (**a**), but not in DLI cells (**b**) when [Ca^2+^] measurements were performed in 1 μM AM251 pre-incubated slices. (**c**–**j**) Effect of 100 nM AM251 on individual boutons in basket (**c**-**f**) and DLI cells (**g**–**j**) in stratum pyramidale (**c**,**d**,**g**,**h**) and in dendritic layers (**e**,**f**,**i**,**j**). The imaged boutons are labelled on the confocal Z-stack maximum intensity projection image (red, biocytin; green, CB_1_), and corresponding [Ca^2+^] transients are shown before (left traces) and after (right traces) AM251 application (**c**,**e**,**g**,**i**). Amplitude values of [Ca^2+^] transients recorded from individual boutons in control condition and after AM251 application are shown in **d**,**f**,**h** and **j**. AM251 significantly increases [Ca^2+^] transients in strata pyramidale (**d**, paired *t*-test, *P*=0.005, *n*=7 boutons) and lucidum (**f**, paired *t*-test, *P*=0.012, *n*=7 boutons) in basket cells. (**k**) AM251 effect on the amplitude of [Ca^2+^] transients at individual cells in strata pyramidale and lucidum/radiatum. Every symbol represents an individual cell. (**l**–**m**) The AM251 effect does not depend on either the peak [Ca^2+^] (**l**) or the relative CB_1_ content of the boutons (**m**). Measurements were performed in stratum pyramidale (circles) and radiatum/lucidum (triangles) for basket (black) and DLI (red) cells, respectively. When the correlation was analysed only for basket cells, no significant effect was seen. (**n**–**o**) Correlation analysis at the level of individual boutons revealed significant correlation between AM251 effect and *G*/*G*_max_ in five out of nine cells (**n**). Each cell is colour coded and open symbols represent individual boutons. There was no correlation between AM251 effect and CB_1_ immunoreactivity in all analysed (*n*=7) cells (**o**). Error bars in **a**,**b**,**d**,**f**,**h**,**j** and **k** represent s.d. Scale bars, 5 μm (**c**,**e**,**g**,**i**). *denotes *P*<0.05.

Many factors could cause the smaller AP-evoked [Ca^2+^] transients in basket cell boutons; the most obvious factors are (1) larger bouton volumes, (2) higher endogenous Ca^2+^ buffering and (3) smaller Ca^2+^ influx in basket cells. Our 3D EM reconstruction data showed that CCK+ boutons were 122% larger in stratum pyramidale than in stratum radiatum (Supplementary Fig. 4d). Different endogenous Ca^2+^ buffering is unlikely, because the decay of the [Ca^2+^] transients did not differ between basket and DLI cells (unpaired *t*-test, *P*=0.72). The smaller Ca^2+^ influx in basket cell boutons can be a consequence of a larger CB_1_-dependent suppression of N-type Ca^2+^ channel function in these cells. Therefore, we examined how tonic, endocannabinoid (eCB)-mediated regulation of presynaptic [Ca^2+^] depends on the recorded cell type and on the CB_1_ content of the boutons. First, we compared the [Ca^2+^] transients recorded in control conditions and in slices pre-incubated with AM251 (1 μM) and found significantly higher transients in basket (Fig. 6a, unpaired *t*-test, *P*<0.001, *n*_control_=28, *n*_AM251_=6), but not in DLI (Fig. 6b, unpaired *t*-test, *P*=0.48, *n*_control_=21, *n*_AM251_=8) cells. We also bath-applied AM251 (0.1 μM) to naive slices after recording [Ca^2+^] transients in control conditions and detected a significant (paired *t*-test, *P*=0.017) increase in basket cells in stratum pyramidale (Fig. 6c,d,k), but not in the dendritic layers (Fig. 6e,f,k). Bath application of 0.1 μM AM251 caused no significant change in [Ca^2+^] transients of DLI cells in either the stratum pyramidale (Fig. 6g,h,k) or the dendritic layers (Fig. 6i–k). In addition, a higher AM251 concentration (2 μM) did not change the amplitude of [Ca^2+^] in DLI cells when measured in the dendritic layers (control *G*/*G*_max_=0.25±0.08, AM251 *G*/*G*_max_=0.28±0.12, *n*=5; effect of 0.1 μM: 12±12% versus effect of 2 μM: 9±14%, *P*=0.74, unpaired *t*-test).

These results reveal a hippocampal layer-specific effect of AM251 on basket cell axon terminals. Furthermore, we performed ‘double recordings’ on bouton strings of individual cells that located in distinct layers and tested the effect of AM251 on the peak [Ca^2+^]. Similarly to individual cell recordings, the effect of AM251 was larger in stratum pyramidale (77±57%) than in dendritic layers (22±26%) when analysed at the level of individual boutons of a given cell (Supplementary Fig. 6a–d) or for single cells (Supplementary Fig. 6e).

*Post hoc* identification of the recorded cells and quantitative determination of their CB_1_-IR allowed us to reveal the lack of significant correlation between the effect of AM251 and the initial [Ca^2+^] (Fig. 6l, Spearman’s *ρ*=−0.48, *P*=0.062, *n*=16) or the CB_1_-IR (Fig. 6m, *ρ*=0.11, *P*=0.7, *n*=15) when all cells were analysed together (blue regression lines). Next, we investigated the correlations between AM251 effect and initial [Ca^2+^] or CB_1_-IR in basket cells only, the cell population where significant AM251 effect was observed, but we still failed to reveal any significant correlation (Fig. 6l, *ρ*=−0.12, *P*=0.77, *n*=9; Fig. 6m, *ρ*=−0.43, *P*=0.29, *n*=8, black regression lines). Finally, the measurements of [Ca^2+^], AM251 effects and CB_1_-IR in individual axon terminals allowed us to investigate the correlations between these parameters at the level of individual boutons within single cells. The AM251 effect showed a negative significant correlation with [Ca^2+^] in 5 out of 9 cells (Fig. 6n, *ρ*-values were −0.81, −0.82, −0.82, 0.14, −0.57, −0.98, −0.91, −0.21 and −0.23). However, when the effect of AM251 was examined as a function of CB_1_-IR, we found no significant correlations in our analysed 7 cells (Fig. 6o, *ρ*-values were 0.079, −0.29, −0.10, 0.77, 0.43, −0.38 and −0.43).

### Cell type- and CB_1_-content-independent effect of CB_1_ agonist

**Figure 7:**
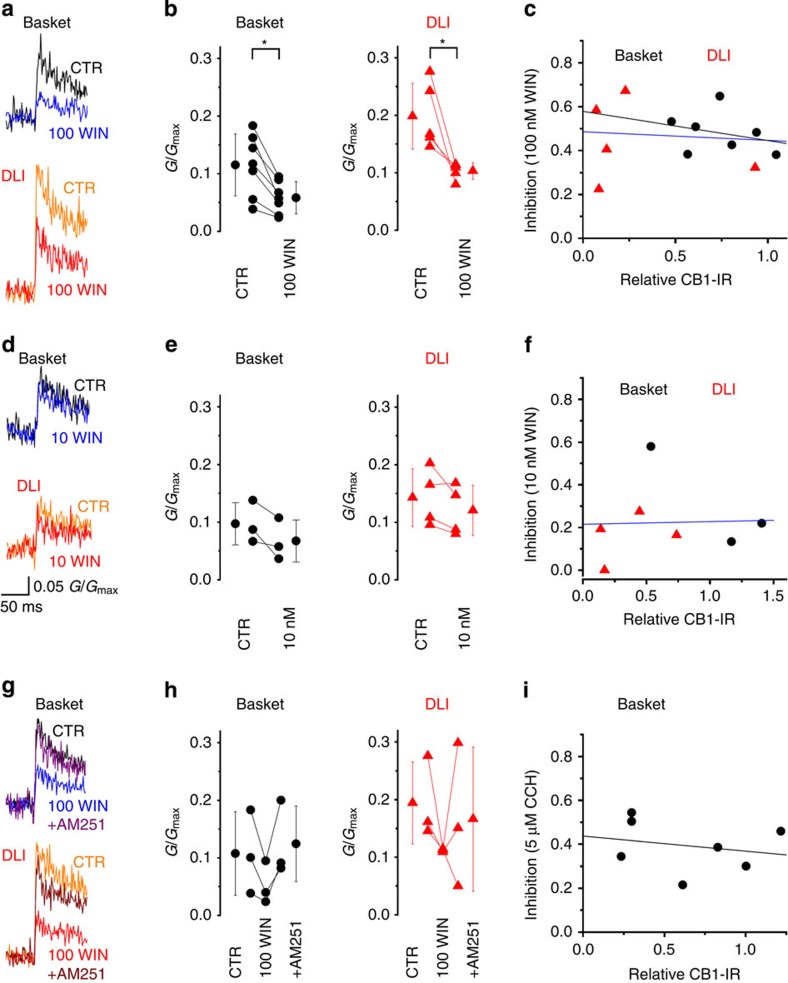
Activation of the CB _1_ significantly decreases [Ca ^2+^ ] transients in axon terminals of basket and DLI cells in the CA3 area. (**a**,**b**,**d**,**e**) Effect of the CB_1_ agonist WIN 55212-2 (10 and 100 nM) on [Ca^2+^] transients in individual basket (in stratum pyramidale) and DLI cells (in dendritic layers). The effect of 10 nM did not reach significance, but that of 100 nM was significant. (**c**,**f**) The WIN 55212-2 effect does not depend on the CB_1_ content of cells when analysis were either performed in all recorded cell (blue regression lines in **c** and **f**) or only for basket cells (black regression line in **c**). (**g**,**h**) The effect of 100 nM WIN 55212-2 can be reversed by the CB_1_ antagonist AM251 (1 μM). (**i**) The effect of 5 μM carbachol did not depend on the CB_1_ immunoreactivity of the cells. Error bars in **b**,**e** and **h** represent s.d. All scales for **a**,**d** and **g** are shown in **d**. *denotes *P*<0.05.

Since AM251 did not have a significant effect on [Ca^2+^] transients in DLI cells, in our final sets of experiments we tested how the CB_1_ agonist WIN55212-2 (WIN) affects [Ca^2+^] transients, and analysed the correlation between the drug effect and the CB_1_ content of the boutons (Fig. 7). The application of 100 nM WIN significantly decreased the AP-evoked [Ca^2+^] transients (Fig. 7a,b) in DLI cells. This effect was concentration dependent; 10 nM caused 16±12% (Fig. 7e, paired *t*-test, *P*=0.16, *n*=4), whereas 100 nM resulted in 44±18% (Fig. 7b, paired *t*-test, *P*=0.027, *n*=5) reduction in DLI cells and was reversed by AM251 (Fig. 7g,h). We also performed the same analysis on basket cells and found a similar concentration-dependent inhibition (10 nM: 31±24%, *P*=0.13, *n*=3, paired *t*-test; 100 nM: 48±9%, *P*=0.003, *n*=7, paired *t*-test; Fig. 7b,e). The effect of WIN did not show a correlation with the CB_1_ content of the boutons either when both cell types were analysed together (Fig. 7c, blue regression line, Spearman’s *ρ*=−0.22, *P*=0.48, *n*=12; Fig. 7f, blue regression line, *ρ*=−0.61, *P*=0.15, *n*=7) or when only basket cells were evaluated (Fig. 7c, black regression line, *ρ*=−0.46, *P*=0.29, *n*=7). Finally, we also revealed a lack of correlation between CB_1_-IR and the carbachol-induced^33^ increased eCB-evoked reduction of [Ca^2+^] transients (Fig. 7i, Spearman’s *ρ*=−0.18, *P*=0.7, *n*=7).

In summary, our experiments clearly show the lack of correlation between the total CB_1_ content of boutons and the degree of CB_1_-mediated control of presynaptic [Ca^2+^] transients.

## Discussion

In the present study, we report the first investigation of the ‘number–function’ relationship for a metabotropic receptor in a small subcellular compartment of a central neuron; CB_1_ on axon terminals of CCK-expressing hippocampal GABAergic INs. To determine the ‘number’ of CB_1_, we quantified LM immunofluorescent labelling using confocal microscopy. EM SDS-FRL was also used as an independent method for providing a quantitative comparison of the CB_1_ density of boutons in distinct layers. Currently, SDS-FRL is widely accepted as the method of choice for quantitative, high-resolution localization of plasma membrane proteins. This method bestows unprecedented sensitivity and high resolution^34^. Unfortunately, a major weakness of SDS-FRL is that it cannot be used for analysing the molecular content of physiologically characterized individual cells due to random fracturing of the tissue. SDS-FRL experiments revealed that axon terminals contain a significantly higher (26% in mice, 31% in rats) density of CB_1_ in stratum pyramidale than in the stratum radiatum. This difference in density is smaller than that (71% in postfixed slices; 49% in perfused mice; 83% in perfused rats) found in the total CB_1_ content of axon terminals in strata pyramidale and radiatum. Performing EM 3D reconstruction from serial ultrathin sections, we found that the surface area of CCK+ boutons was 113% higher in stratum pyramidale than in the stratum radiatum. This finding is consistent with recent studies showing that axon terminals of Schaffer collateral-associated INs are smaller than those of basket cells in the CA1 area^25^,^28^. When we measured the total CB_1_ content of identified basket and DLI axon terminals, differences in mean CB_1_-IR between these subtypes were revealed. This is again consistent with the finding of Dudok *et al.*^25^ in CA1 using LM immunofluorescent labelling and super-resolution analysis. Our results also showed a robust within-cell type variability in CB_1_-IR of the recorded and analysed basket (CV=0.35) and DLI (CV=0.69) cells. If the CB_1_ content of a presynaptic bouton depends on the identity of postsynaptic target cell, then a potential reason for the higher variability in DLI cells might be that these INs differentially innervate distinct types of PCs projecting to specific brain regions^35^, or they may form synapses with a larger fraction of GABAergic INs as postsynaptic targets^28^.

To estimate the ‘function’ of the CB_1_ activation, two-photon Ca^2+^ imaging was performed in acute hippocampal slices at the level of individual axon terminals of CCK+ basket and DLI cells. A previous study from our laboratories provided a quantitative understanding of how the presynaptic [Ca^2+^] affects release at these GABAergic axon terminals^18^ and showed that tonic CB_1_ activation exerts its effect mainly through the reduction of N-type Ca^2+^ channel function. Thus, knowing the relationship between [Ca^2+^] and release, and that CB_1_ only affects Ca^2+^ influx, we used presynaptic [Ca^2+^] transients as an approximation of presynaptic release. Our experiments revealed a 107% larger presynaptic [Ca^2+^] transient in DLI compared with basket cell axon terminals in control conditions. This difference is partly due to the larger CB_1_-mediated reduction of [Ca^2+^] in basket cells (estimated by the effect of AM251). However, when the amplitudes of [Ca^2+^] are compared in the presence of AM251 between basket and DLI cells, an ∼45% larger transient is still observed (data pooled from the steady-state and wash-in experiments, Fig. 6a,b,k). This can be explained by more pronounced Ca^2+^ buffering^36^, a larger bouton volume^28^ or a smaller Ca^2+^ influx in basket cells. Although, we have not directly tested the Ca^2+^ buffering capacity in the axons of the two different cell types, the similar decay kinetic of the [Ca^2+^] transients is inconsistent with large differences in buffering^36^. It is worth noting that some of the DLI cells may contain calbindin-D28k^28^,^29^,^37^, a calcium-binding protein that should increase the buffer capacity of those DLI cells. However, a larger endogenous buffering would reduce, rather than increase the measured [Ca^2+^] transient in DLI cells. Our EM 3D reconstructions revealed that the mean volume of boutons in stratum pyramidale was ∼120% larger than that in stratum radiatum. Calculating the total fluxed Ca^2+^ from the [Ca^2+^] and bouton volume indicated that ∼65% more Ca^2+^ enters the terminals of basket cells upon an AP. This value is very similar to the ratio (1.5) of total AZ areas in boutons in strata pyramidale and radiatum, indicating a similar Ca^2+^ channel density in the AZs of these two different cell types.

Our results demonstrated that basket cell axon terminals contain more CB_1_, have smaller initial [Ca^2+^] and a larger tonic eCB-mediated reduction of [Ca^2+^] than DLI cells. However, it was surprising to see the lack of correlation between CB_1_-IR and the effect of drugs that modulate CB_1_ function (AM251, WIN55212-2 and carbachol). The two most likely explanations of these results are as follows.

(1) Tonic eCB-mediated reduction of [Ca^2+^] might be primarily determined by the concentration of the ligand, and not the number of receptors. Our results would then predict a higher eCB concentration in stratum pyramidale compared with the dendritic layers. The eCB causing this effect is either 2-arachidonoyl glycerol (2-AG) or anandamide^38^,^39^,^40^. The 2-AG-synthesizing enzyme (diacylglycerol lipase-α) has been found mainly in spines of PCs, resulting in a higher concentration in dendritic layers compared with stratum pyramidale^41^. This regional distribution of diacylglycerol lipase-α would predict an opposite 2-AG concentration gradient: high in the dendritic layers and low in stratum pyramidale. The concentration of 2-AG, however, does not only depend on its synthesizing enzyme, but also its degrading pathway. The hydrolyzing enzyme of 2-AG (monoacylglycerol lipase-α) is also concentrated in the dendritic layers of the hippocampus, where it is located mainly in glutamatergic axons^42^. In addition, monoacylglycerol lipase-α was shown to be present in astrocytes^43^, which has less processes in stratum pyramidale compared with the dendritic layers^44^, indicating that glia cells could also play a role in the degradation of 2-AG in the dendritic layers. However, due to the highly lipophilic nature of 2-AG, it is very unlikely that it would effectively diffuse hundreds of microns from the dendritic layers to the stratum pyramidale. The potential role of anandamide is even more uncertain: the synthesizing enzyme (*N*-acyl phosphatidylethanolamine-specific phospholipase D) is located on presynaptic glutamatergic axons resulting in a high density in the dendritic layers^45^, whereas one of its degrading enzymes (fatty acid amide hydrolase) is present at high concentrations in stratum pyramidale^42^.

(2) Another possible explanation is a more efficient coupling of CB_1_ to N-type Ca^2+^ channels in basket cells. Factors that determine the ‘coupling efficiency’ are largely unknown, but a potential way of conceptualizing it might be that the ‘functionally relevant’ CB_1_s could have a special location within the boutons. Where could these ‘special’, ‘functionally relevant’ receptors be? Here we provided direct evidence for molecular-scale colocalization of the N-type Ca^2+^ channels with CB_1_ probably within the AZ. Our SDS-FRL reactions clearly demonstrated the presence of CB_1_ in AZs in addition to peri- and extrasynaptic locations, in agreement with the results of a previous study applying postembedding immunogold localization^24^. Our data also revealed heterogeneity in the CB_1_ content of AZs; some Rim1/2-positive AZs were strongly (Fig. 3b; Supplementary Fig. 2b) labelled, whereas some were apparently immunonegative (Fig. 3c; Supplementary Fig. 2c). This raises the possibility that the degree of tonic eCB-mediated modulation of presynaptic [Ca^2+^], and consequently neurotransmitter release, might depend on the amount of CB_1_ in the presynaptic AZ close (tens of nanometres) to N-type Ca^2+^ channels. The different amounts of CB_1_ in AZs could be achieved by the rapid diffusion of receptors in and out of synapses as shown with single-molecule tracking after agonist-induced desensitization^46^.

The above mentioned two possibilities are of course not mutually exclusive; if CB_1_s in the AZs have a special role, there could still be differences in eCB concentrations in distinct hippocampal layers and synapses.

Furthermore, we cannot rule out the possibility that phasic eCB-mediated regulation of release (for example, during depolarization-induced suppression of inhibition or inhibitory long-term depression) depends on the total bouton CB_1_ content. A scenario might be envisaged in which, similar to the pure synaptic versus synaptic plus extrasynaptic activation of postsynaptic AMPA and GABA_A_ receptors, the population of presynaptic CB_1_ that mediates the regulation of release might change as a function of postsynaptic activity.

To directly test these models, a novel quantitative localization approach needs to be developed, as neither of the widely used preembedding immunogold or immunofluorescent techniques are capable of visualizing synaptic receptors without antigen retrieval, and SDS-FRL cannot be used on functionally characterized axon terminals.

## Methods

### Slice preparation

Acute 250-μm-thick horizontal hippocampal slices were cut from 19.3±2.5-day-old (P15–P27) transgenic mice (*n*=128) from both sexes expressing DsRed fluorescent protein under the control of the cholechystokinin promoter (*CCK-BAC/DsRedT3* mouse line)^32^. Animals were handled in accordance with the Hungarian Act of Animal Care and Experimentation (1998, XXVIII, section 243/1998) and with the ethical guidelines of the Institute of Experimental Medicine Protection of Research Subjects Committee. Mice were anaesthetized with isoflurane, then the brains were quickly removed and placed into an ice-cold solution containing (in mM): sucrose, 204.5; KCl, 2.5; NaHCO_3_, 26; CaCl_2_, 0.5; MgCl_2_, 5; NaH_2_PO_4_, 1.25; glucose, 10; bubbled with 95% O_2_ and 5% CO_2_. Hippocampal slices were cut using a Leica vibratome (Leica VT1200S; Leica Microsystems), then incubated in an interface-type holding chamber filled with 36 °C ACSF containing (in mM): NaCl, 126; KCl, 2.5; NaHCO_3_, 26; CaCl_2_, 2; MgCl_2_, 2; NaH_2_PO_4_, 1.25; glucose, 10; saturated with 95% O_2_ and 5% CO_2_. Recordings were carried out using the same ACSF solution at 22 °C. Slices were kept up to 6 h in the holding chamber prior to use.

### Electrophysiological recordings and two-photon imaging

Two-photon imaging experiments were performed with a Femto2D (Femtonics Ltd.) laser scanning microscope, using a MaiTai femtosecond pulsing laser (Spectra Physics) tuned to 810 nm. Cells were visualized using oblique infrared illumination and a water immersion lens ( × 25, 1.05 numerical apperture, Olympus or × 25, 1.1 numerical apperture, Nikon). CCK-containing INs in the CA3 region of hippocampus expressing DsRed were identified based on their red fluorescence. Whole-cell recordings were conducted in current-clamp mode using a MultiClamp 700A amplifier (Molecular Devices). Electrophysiological traces were filtered at 3 kHz and digitized online at 20 kHz. Patch pipettes were pulled (Zeitz Universal Puller; Zeitz-Instrumente Vertriebs) from thin-walled borosilicate glass capillaries with an inner filament (1.5 mm outer diameter, 1.17 mm inner diameter; Harvard Apparatus). Pipette resistance was 5–7 MΩ. The intracellular solution contained (in mM): K-gluconate, 130; KCl, 5; MgCl_2_, 2; creatine phosphate, 10; HEPES, 10; Na_2_ATP, 2; Na_2_GTP, 1; biocytin, 7, pH was adjusted to 7.3; the osmolarity was 280–290 mOsm. Before the recordings, 100 μM Fluo-5F (*K*_D_∼2.3 μM, Molecular Probes) and 25 μM Alexa Fluor 594 (A594, Molecular Probes) were added to each aliquot of intracellular solution. INs were held between −60 and −65 mV with a maximum of −200 pA d.c. current injection. Pairs of APs at 10 Hz were evoked every 30 s with 1.5–2-ms-long depolarizing (1–1.2 nA) current pulses. Two-photon imaging and electrophysiological data were analysed with a Matlab-based software (MES, Femtonics Ltd). Only the first AP-evoked [Ca^2+^] transients were analysed in this study. Drugs were applied using a recirculation system with a peristaltic pump, where the total volume was ∼6 ml and the solutions were equilibrated with 95% O_2_ and 5% CO_2_. AM251 (Tocris) was dissolved in dimethylsulphoxide (DMSO) to reach a 10-mM stock solution. Experiments were carried out to show that DMSO does not affect the amplitude of [Ca^2+^] transients at 0.1% final bath concentration. The DMSO concentration was 0.01% or 0.001% during AM251 pre-incubation (1 μM) and bath application (0.1 μM) experiments, respectively. WIN 55212-2 was dissolved in 0.1 M HCl solution to reach 20 mM concentration. The 5 mM HCl solution, which is 1,000-fold higher than the highest concentration of HCl in the WIN solution used in the experiments, did not change the pH of the ACSF. Carbachol (20 mM stock) was dissolved in distilled water. All drugs were purchased from Sigma unless indicated otherwise.

For detailed description of the imaging experiments and calculation of *G*/*G*_max_, see Holderith *et al.*^26^ and Szabo *et al.*^18^ Briefly, after an hour of loading period, a 20-min ‘control’ scanning period was performed during which ∼10–15 boutons at 30–60 μm depth were scanned (500-ms-long line-scans were applied with a 1-kHz sampling rate) each twice with a 30-s interval. The same 20-min scanning period was repeated on the same boutons after a 15- or 30-min drug equilibration period. The amplitude of [Ca^2+^] transients went through a gradual decline during the ∼2-h imaging session in ACSF; therefore all drug effects were normalized to their corresponding reduction in ACSF. Control recordings in ACSF were intermingled with the drug experiments, and the reduction in the peak amplitude of [Ca^2+^] in ACSF was calculated for each block/series of pharmacology experiments (6–8 weeks per block). The amplitudes in ACSF were reduced to 92.9±15% (*n*=11, first set of experiments) and 88.9±18% (*n*=6, second set of experiments) after 15 min, and 87.7±14.3% (*n*=4) after 30 min. In some experiments the scanning period was lengthened to 35–55 min when two or three hippocampal layers were imaged. For those experiments, we performed ACSF controls at time points of 15, 35 and 55 min, and the amplitudes were reduced to 91.5±7.5% (*n*=3), 82.2±11.9% (*n*=3) and 72.7±4.8% (*n*=3) of control, respectively. Throughout the figures, the [Ca^2+^] transient traces are not modified, but the summary data are normalized to these reductions. The effect of drugs for individual boutons/cells was calculated by dividing the peak amplitude value of [Ca^2+^] transients (for a bouton: measured from the average of two individual traces; for cells: measured from the average of all individual traces of boutons in that cell) in the presence of the drug with that in control condition (peak measured within a time window of 4–11 ms after AP initiation), and then this ratio was normalized to the decline observed in ACSF (see above). The [Ca^2+^] transients of individual cells did not depend on the somatic AP full-width at half-maximal amplitude, AP peak amplitude, the access resistance, the holding potential (between −54 and −68 mV), the holding current (from 0 to −200 pA) or the ‘baseline’ green fluorescence measured after 60 min of dye loading (Supplementary Fig. 7a–f). In addition, no correlation was found between the distance of the imaged bouton string from the soma and the amplitude or the decay of [Ca^2+^] transients, or the amplitude of the Alexa Fluor 594 fluorescence (Supplementary Fig. 7g–i).

### Fluorescent immunohistochemistry

After the imaging experiments, slices were placed in a fixative containing 4% paraformaldehyde (PFA) and 0.2% picric acid in 0.1 M phosphate buffer (PB; pH=7.4) at 4 °C for >12 h, and then were washed in PB several times, embedded in agarose (2%) and re-sectioned at 60–80 μm thickness. Sections were washed with PB and blocked in normal goat serum (NGS; 10%) made up in Tris-buffered saline (TBS, pH=7.4). CB_1_ was visualized using a rabbit anti-CB_1_ antibody (Cayman Chemical Company, Cat. No. 10006590), which was raised against amino acids (aa.) 461–472 of the human CB_1_ receptor, diluted in 1:1,000 in TBS containing 0.1% Triton. For some immunoreactions on perfusion-fixed tissue sections, a guinea pig anti-CB_1_ (1:500; Frontier Institute Co. Ltd, Cat. No. CB_1_-GP-Af530) antibody was also used. The epitope of this antibody (aa. 443–473 of the mouse CB_1_ protein)^47^ overlaps with that of the rabbit antibody. Identical labelling of the two CB_1_ antibodies was confirmed by double-labelling experiments. The specificity of the CB_1_ immunoreactions was confirmed using *CB1*^*−/−*^ and *CB1*^*+/+*^ mice^48^. After several washes in TBS, Alexa488-conjugated goat anti-rabbit immunoglobulin (Ig)G (1:500; Life Technologies) or Cy3-conjugated donkey anti-guinea pig IgG (1:1,000; Jackson ImmunoResearch Laboratories) was used to visualize the CB_1_ immunoreaction, while Cy3-conjugated streptavidin (1:500; Jackson ImmunoResearch Laboratories) was used to visualize the biocytin. Sections were then mounted on slides in Vectashield (Vector Laboratories). Images were acquired using an Olympus FV1000 confocal microscope with either a × 10, × 20 or a × 60 objective. Cells were identified as basket, mossy fiber-associated, DLI and perforant-path-associated cells based on their axonal arborization^20^,^27^,^28^,^29^,^30^. Mossy fiber-associated and perforant-path-associated cells were discarded from the analysis because of the low number of recordings. Only CB_1_-immunopositive cells were included in the study.

### Evaluating the CB_1_ content of boutons in hippocampal layers

Immunohistochemical reaction for CB_1_ was performed on *in vitro* slices that were immersion fixed and re-sectioned after the recording and imaging session and on sections from perfusion-fixed male rat (P30-36, 34.5±2.5-day-old animals, *n*=4) and *CCK-BAC/DsRedT3* male mouse (P20-25, 23.2±2-day-old animals, *n*=5) brains. Confocal Z-image stacks (with the same laser power, photomultiplier settings, pinhole size and dwell time) were acquired between 5 and 10 μm depth from the tissue surface with a 0.5-μm step size in strata pyramidale, lucidum, radiatum and oriens in the distal CA3 region. In each Z-image stack, 32–40 CB_1_-positive boutons were chosen randomly at single confocal layers and circular/elliptic regions of interest (ROIs) were placed over them (usually 8–10 CB_1_-positive boutons and two background ROIs in four optical layers) using the FV1000-ASW1.6 software (Olympus Corporation). Special care was taken to select boutons in focus, and boutons were discarded if additional fluorescence coming from neighbouring boutons affected the measurement (Supplementary Fig. 8). The integral of the CB_1_ signal was measured in every ROI, including CB_1_-positive boutons and surrounding background areas. The background-subtracted CB_1_ intensity of the boutons was then calculated. To compare different hippocampal regions within a slice, mean CB_1_ intensity values of strata pyramidale, lucidum and oriens were normalized to those obtained in the stratum radiatum.

### Evaluating the CB_1_ content of individual cells

The relative CB_1_-IR (obtained with the rabbit anti-CB_1_ antibody) of whole-cell recorded GABAergic INs was calculated as follows: confocal Z-image stacks were acquired in those hippocampal layers where the Ca^2+^ imaging took place (usually the same region where the main axonal arbor of a cell was) in addition to stratum pyramidale. The image stacks were acquired in the same way as described above. Biocytin-filled boutons (18±6.8 boutons; not the ones that were *in vitro* Ca^2+^ imaged) were identified in the imaged hippocampal layer, and 32 biocytin-unlabelled boutons were randomly selected in the stratum pyramidale. CB_1_ intensity was measured using ROIs placed over boutons, the average of CB1 intensity integrals were calculated for biocytin-containing and -lacking boutons. The strength of the CB_1_-IR of a cell was normalized to the average CB_1_ reactivity of randomly chosen boutons in the stratum pyramidale. CB_1_-IR values used for individual bouton analysis were not normalized (Figs 5j and 6o).

### SDS-digested freeze-fracture replica-labelling (SDS-FRL)

Male Wistar rats (P30–P46, 40.8±6.6, *n*=5), *CCK-BAC/DsRedT3* mice (P19–P25, 22.9±2.5, *n*=2 female, *n*=7 male), *CB*_*1*_^*+/+*^ (P18, P26, *n*=2 female) *CB*_*1*_^*−/−*^ (P18, *n*=2 female)^48^, *Cav2.2*^*+/+*^ (P18, *n*=1) and *Cav2.2*^*−/−*^ (9 months old, *n*=1)^49^ mice were deeply anaesthetized and transcardially perfused with an ice-cold fixative containing 2% PFA and 15*v*/*v*% picric acid in 0.1 M PB for 15 min. Coronal sections of 80 μm thickness were cut from the forebrain with a vibratome and were cryoprotected in 30% glycerol. Replicas were prepared as described previously^50^,^51^. Briefly, small blocks from the CA3 area were frozen in a high-pressure freezing machine (HPM100, Leica Microsystems) and fractured at −135 °C in a freeze-fracture machine (BAF060, Leica Microsystems). The fractured tissue surfaces were coated with thin layers of carbon (5 nm), platinum (2 nm) and carbon (20 nm). Tissue debris was digested from the replicas in a solution containing 2.5% SDS and 20% sucrose in TBS at 80 °C overnight. Following several washes in TBS containing 0.05% bovine serum albumin (BSA), replicas were blocked in TBS containing 2.5–5% BSA for 1 h, then incubated overnight in the same blocking solution containing primary antibodies. The following primary antibodies were used: rabbit anti-CB_1_ antibody (1:500, Cayman Chemical, Cat. No. 10006590), guinea pig anti-CB_1_ (1:100, Frontier Institute Co. Ltd, Cat. No. CB_1_-GP-Af530; used for quantification), guinea pig anti-VGAT (1:2,000, Synaptic Systems, Cat. No. 131 004), rabbit anti-Rim1/2 (1:2,000; Synaptic Systems, Cat. No. 140 203), rabbit anti-Cav2.2 (1:400, Synaptic Systems, Cat. No. 152 303), guinea pig anti-Cav2.1 (1:100, Frontier Institute Co. Ltd, Cat. No. VDCCa1A-GP-Af810) and rabbit anti-Cav2.1 (1:500, Synaptic System, Cat. No. 152 203). Replicas were then incubated for 2 h in TBS containing 5% BSA and goat anti-rabbit IgGs coupled to 5, 10 or 15 nm gold particles (1:100; British Biocell International Ltd) or to 6 nm gold particles (1:30; AURION Immuno Gold Reagents & Accessories) and goat anti-guinea pig IgGs coupled to 10 or 15 nm gold particles (1:50 or 1:100; British Biocell or 1:30; AURION). Finally, replicas were rinsed in TBS and in distilled water before being picked up on copper parallel-bar grids coated with pioloform. Specimens were analysed with a transmission EM (JEM-1011, JEOL Ltd). All antibodies used in this study recognized intracellular epitopes and consequently were visualized on the P-face. The specificity of the CB_1_ immunolabelling was tested on replicas in *CB*_*1*_^*−/−*^ and *CB*_*1*_^*+/+*^ mice. In single-labelling experiments, immunogold particles for CB_1_ were abolished from axon terminals in every layer of the CA3 in the *CB*_*1*_^*−/−*^ tissue (*n*=2 mice). Double-labelling reactions for CB_1_ using two different anti-CB_1_ antibodies resulted in double-labelled axon terminals in *CB*_*1*_^*+/+*^ mice (Fig. 2h), while on replicas from *CB*_*1*_^*−/−*^ mice no labelled profiles were found (Fig. 2i). Furthermore, CB_1_ labelling showed extensive colocalization with VIAAT in *CB*_*1*_^*+/+*^ mice (31 CB_1_-positive out of 60 VIAAT-positive boutons; Fig. 2j), but in *CB*_*1*_^*−/−*^ mice all VIAAT terminals (0 out of 53) were immunonegative for CB_1_ (Fig. 2k). For quantitative analysis, electron micrographs of presynaptic axon terminals labelled for CB_1_ were taken (*n*=552 boutons in 4 rats, *n*=705 boutons in 6 CCK-BAC/DsRedT3 mice) from strata oriens, pyramidale, lucidum and radiatum of the distal CA3 area with a Cantega G2 camera (Olympus Soft Imaging Solutions GmbH) at × 10.000–25.000 magnifications. The nonspecific background labelling was measured on E-face structures surrounding the measured P-faces. Gold particle counting and area measurements were performed with iTEM software (Olympus Soft Imaging Solutions). The specificity of the Cav2.2 immunolabelling was confirmed using tissue derived from *Cav2.2*^*−/−*^ mice, where immunogold particles for Cav2.2 were mostly abolished from P-face sections of axon terminals attached to somatic E-face membranes (0–3 gold particles in 189 profiles, *n*=2 animals), whereas in *Cav2.2*^*+/+*^ mice these structures were strongly labelled for Cav2.2 (0–15 gold particles in 301 profiles, *n*=2 animals). In addition, in *Cav2.2*^*+/+*^ mice, excitatory axon terminals (identified based on the presence of an AZ facing a postsynaptic density on E-faces) contained stronger Cav2.2 labelling (0–7 gold particles in 27 profiles), compared with *Cav2.2*^*−/−*^ mice (0–3 gold particles in 51 profiles). To quantify the CB_1_, Rim1/2, Cav2.2 and Cav2.1 subunit densities on axon terminals (for Fig. 3d,f,h; Supplementary Figs 2f and 3b,e,i), electron micrographs of PC somatic E-face membranes with attached P-face axon terminal fragments were taken at × 10,000–25,000 magnification from stratum pyramidale. These attached P-face profiles showed large variability in sizes. We have restricted our analysis to those profiles that had an area >0.1 and <0.21 μm^2^, corresponding to the range of AZ sizes obtained from our EM 3D reconstructions. The gold particle densities were then calculated in these P-face membranes without assuming that the entire membrane is an AZ. The AZs of CB_1_-positive boutons do not contain an elevated density of intra-membrane particles, therefore their delineation based on morphological criteria is not possible. Molecules that are confined to the AZ should therefore be used in identifying AZs. Here we used Rim1/2 immunolabelling for this purpose and delineated potential AZs in our figures for illustrative purposes only. However, we refrain from performing quantitative analysis of, for example, CB_1_-IR in AZs, due to uncertainties in determining the borders of the AZs.

The colocalization of Rim1/2 and Cav2.2 was not possible because both primary antibodies were raised in rabbit. Here we provided indirect evidence for the potential enrichment of Cav2.2 in AZs in the following way. Using Rim1/2 labelling, we demonstrated that 23.6% of the P-face membrane fragments fractured to large E-face somatic membranes contain AZs. In Cav2.1 and Cav2.2 double labelling, this proportion was almost identical (21.7%). The quantitative analysis of these double-labelling reactions revealed that these two Ca^2+^ channels are almost fully exclusive: 92% of the fractured membranes had either Cav2.1 or Cav2.2 labelling. We also delineated Cav2.2-rich areas of the boutons for illustrative purposes in our figures, but we have no evidence that these areas fully overlap with the AZs.

### Three-dimensional EM reconstruction of boutons

Three CCK-BAC/DsRedT3 mice (P19, P19 and P23, males) were perfused with fixatives containing 2% PFA and 0.5% glutaraldehyde in 0.1 M sodium acetate buffer (pH=6) for 2 min, followed by a 45-min perfusion with a fixative containing 2% PFA and 0.5% glutaraldehyde in 0.1 M sodium borate buffer (pH=9). The brains were left in the skull for 24 h at 4 °C before removal. Horizontal sections (60 μm thick) were cut from the ventral hippocampus with a vibratome, followed by several washes in PB before treatment with 1% sodium borohydrate in PB for 30 min. Sections were then thoroughly washed in PB and TBS, followed by blocking in TBS containing 10% NGS and incubation in mouse anti-CCK antibody (#9303 NIH, CURE/Digestive Disease Research Center, University of California Los Angeles); diluted 1:1,000 in TBS containing 2% NGS and 0.05% Triton X-100 at room temperature overnight. After several washes in TBS, sections were incubated in biotinylated goat anti-mouse secondary antibody in TBS. The reaction was then visualized with the avidin–biotin–horseradish peroxidase complex and 3′3-diaminobenzidine tetrahydrochloride. Sections were then postfixed in 1% O_s_O_4_, stained in 1% uranylacetate, dehydrated and embedded into Durcupan. Blocks containing the strata pyramidale and radiatum of the distal CA3 area were re-embedded, and long series of ultrathin sections were cut for EM. Series of digital images of randomly chosen immunopositive boutons were taken from both layers. Boutons were reconstructed in 3D using the Reconstruct software ( http://synapses.clm.utexas.edu/) and the following ultrastructural parameters were measured: the surface and the volume of the boutons, the number and volume of mitochondria in the boutons, the number of AZs and the area of the AZs. The volume of the terminals was calculated by deducing it with the total mitochondria volume.

### Data analysis and statistical tests

Data were plotted using OriginPro 9.0 (OriginLab Corporation). Statistical tests were performed with Statistica 11 (Scientific Computing). Shapiro–Wilk normality tests showed that the data sets were not different from normal distributions, and therefore parametric statistical tests (paired and unpaired *t*-test, randomized block design or factorial ANOVA with Tukey’s *post hoc* test) were used as indicated in the manuscript. Spearman’s rank-order correlation was used to assess correlations between variables if the sample size was small (<30; Pearson’s correlation gave qualitatively same results in every cases). For larger sample sizes (*n*>70; Supplementary Fig. 7), Pearson’s correlations were used if the data set did not deviate from a normal distribution. Results were considered significant at *P*<0.05. Data are presented as mean±s.d. throughout the manuscript.

## Additional information

**How to cite this article:** Lenkey, N. *et al.* Tonic endocannabinoid-mediated modulation of GABA release is independent of the CB_1_ content of axon terminals. *Nat. Commun.* 6:6557 doi: 10.1038/ncomms7557 (2015).

## Supplementary Material

Supplementary Information Supplementary Figures 1-8
