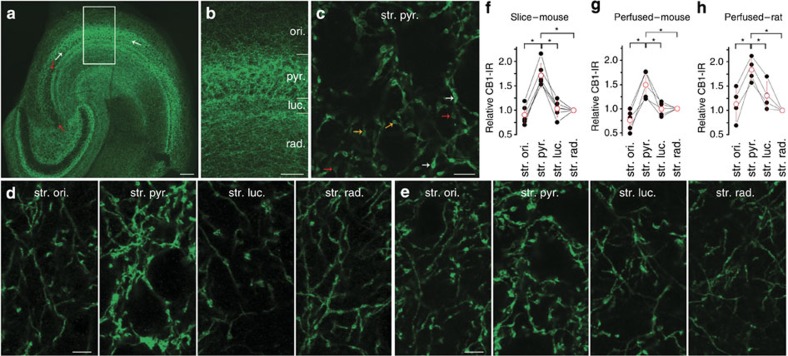# Corrigendum: Tonic endocannabinoid-mediated modulation of GABA release is independent of the CB_1_ content of axon terminals

**DOI:** 10.1038/ncomms9226

**Published:** 2015-08-26

**Authors:** Nora Lenkey, Tekla Kirizs, Noemi Holderith, Zoltán Máté, Gábor Szabó, E. Sylvester Vizi, Norbert Hájos, Zoltan Nusser

Nature Communications 6, Article number: 6557 10.1038/ncomms7557 (2015); Published: 04202015; Updated: 08262015.

In Fig. 1g of this Article, three data series were inadvertently omitted from the graph while the final version of the manuscript was prepared for submission. The correct version of Fig. 1 appears below.

## Figures and Tables

**Figure 1:**