# Supplementary material for: Tonic endocannabinoid-mediated modulation of GABA release is independent of the CB1 content of axon terminals
Source: Nat Commun. 2015 Apr 20;6:6557. doi: 10.1038/ncomms7557 (PMC4413030; doi:10.1038/ncomms7557)
Supplement: Supplementary Information — Supplementary Figures 1-8 [file ncomms7557-s1.pdf]

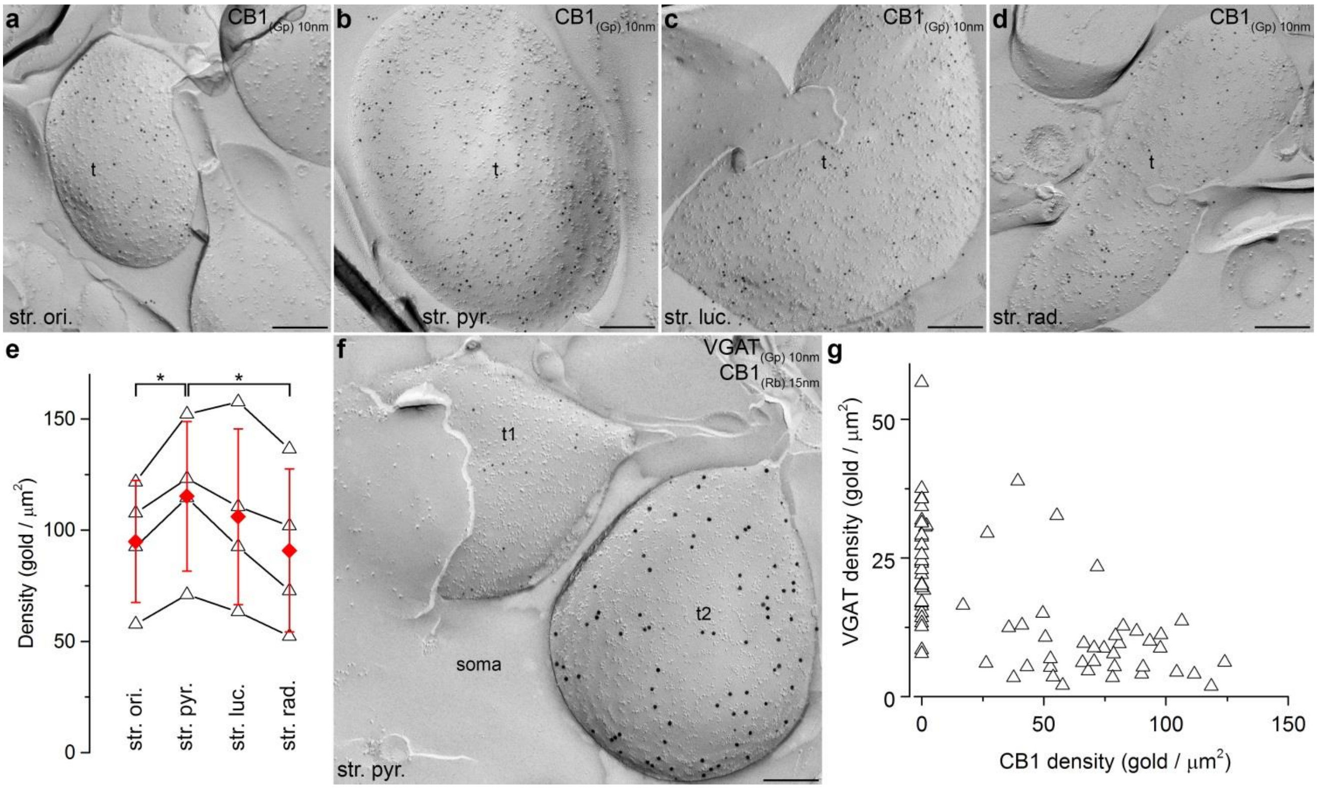

Supplementary Figure 1. SDS-FRL localization of CB_1 in the distal CA3 area of the rat hippocampus. (**a-d**) Axon terminals (t) in stratum pyramidale (**b**) show stronger immunolabeling for CB_1 than those in strata oriens (**a**), lucidum (**c**) and radiatum (**d**). (**e**) Densities of gold particles labeling CB_1 in distinct hippocampal layers. Randomized block design ANOVA followed by Tukey post-hoc test revealed significant differences between strata pyramidale and oriens ($p = 0.03$) and between strata pyramidale and radiatum ($p = 0.012$). Red diamonds represent the mean ± s.d. ($n = 4$). (**f**) A double labeling experiments for CB_1 (15 nm gold) and the vesicular GABA transporter (VGAT, 10 nm gold) demonstrates that the CB_1-immunopositive P-face profile is a GABAergic axon terminal (t2). Note that not all VGAT labeled terminals contain CB_1 (t1). (**g**) Background-subtracted density values for CB_1 and VGAT in VGAT-immunopositive axon terminals are shown. Each symbol represents an individual bouton ($n = 1$ rat). str. ori.: stratum oriens, str. pyr.: stratum pyramidale, str. luc: stratum lucidum, str. rad.: stratum radiatum, gp: guinea pig, rb: rabbit. Scale bars: 200 nm.

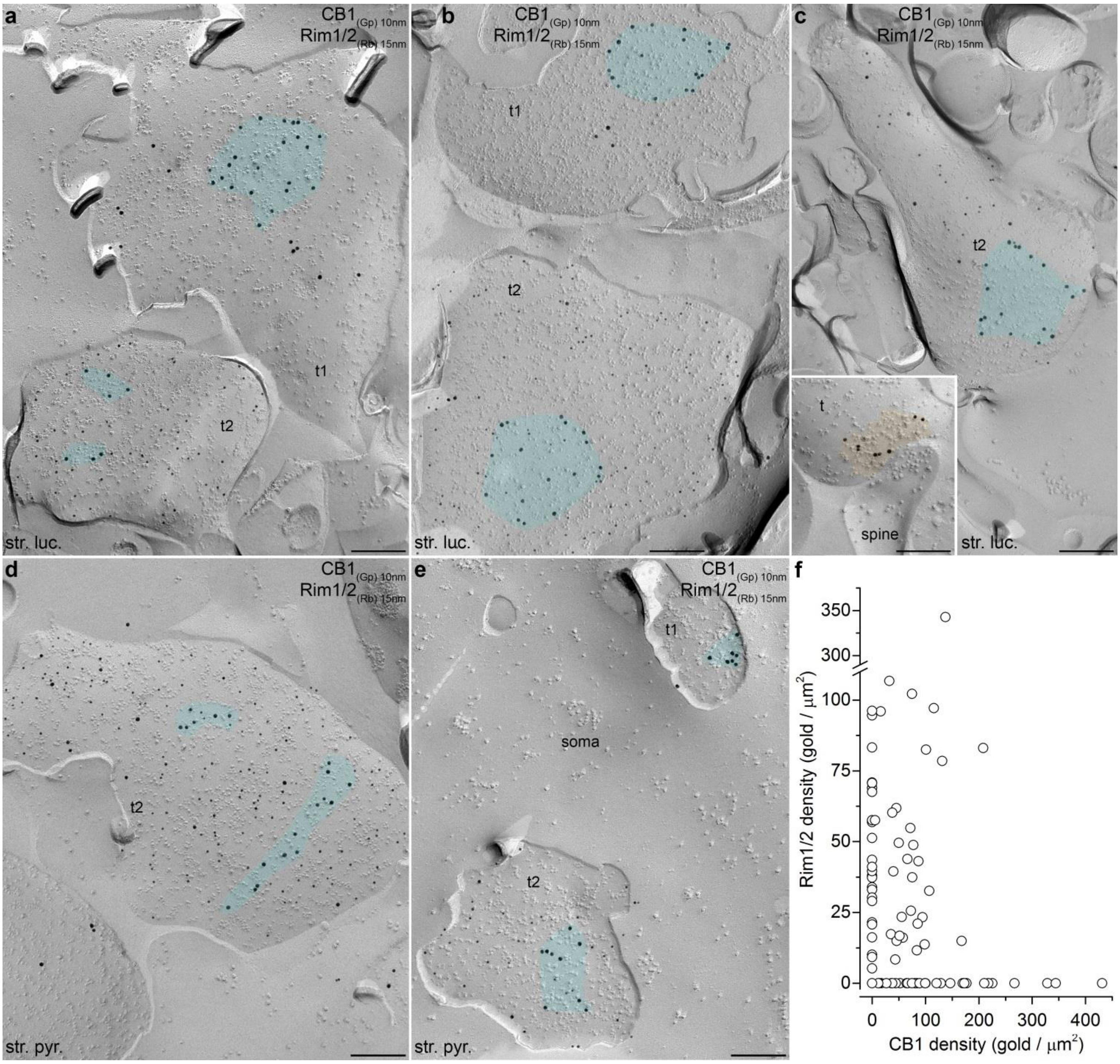

Supplementary Figure 2. Variability in the CB_1 content in active zones (AZ) of CB_1-immunoreactive axon terminals in the rat hippocampus. (**a-e**) Axon terminals contain a variable number of immunogold particles for CB_1 (10 nm gold, t2) in their AZs (blue, labeled by Rim1/2 with 15 nm gold). Around the CB_1-positive axon terminals, CB_1-negative boutons (t1, presumed parvalbumin-positive basket cell boutons) also contain Rim1/2-positive (15 nm gold) AZs. P-face membrane fractures of CB_1 containing (t2, **e**) and lacking (t1, **e**) axon terminals are attached to the E-face membrane of a PC soma. Both of these attached P-face membranes contain an AZ. The AZ of an excitatory terminal (orange) also contains gold particles labeling Rim1/2, but not CB_1 (inset of **c**). (**f**) Background-subtracted densities for CB_1 and Rim1/2 measured in P-face axon terminal fragments attached to the E-face somatic membranes. Every symbol represents a P-face section (only axon terminal sections with the area of 0.010-0207 μm^2 were quantified, n = 1 rat). str. pyr.: stratum pyramidale, str. luc: stratum lucidum, gp: guinea pig, rb: rabbit. Scale bars: 200 nm.

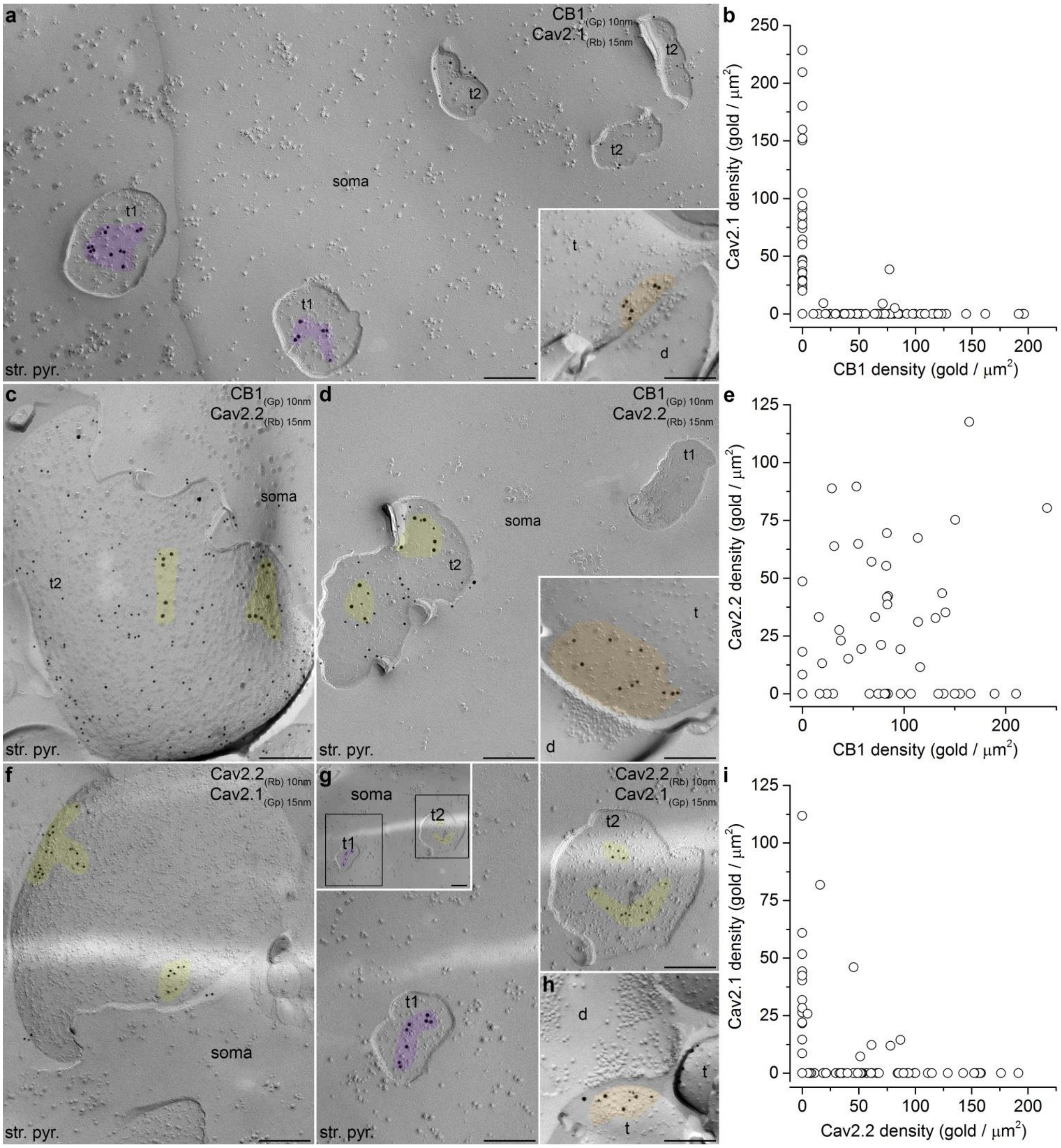

Supplementary Figure 3. CB_1-immunopositive axon terminals contain the Cav2.2, but not the Cav2.1 subunit in the rat hippocampus. (**a**) CB_1 (10 nm gold, t2) or Cav2.1 subunit (15 nm gold, t1) containing P-face axon terminal fragments are attached to a somatic plasma membrane. The active zone (AZ) of an excitatory axon terminal contains Cav2.1 subunit (orange, inset). (**b**) Background-subtracted gold particle densities for CB_1 and Cav2.1 subunit of P-face axon terminal sections attached to the E-face somatic membranes. (**c-d**) CB_1-immunoreactive boutons contain Cav2.2 subunit. A perisomatic axon terminal (t2 in **c**) and a P-face section of an axon terminal (t2 in **d**) attached to somatic E-face membrane are co-labeled for CB_1 (10 nm gold) and Cav2.2 subunit (15 nm gold). An excitatory axon terminal contains only Cav2.2 subunit, but not the CB_1 (orange, inset in **d**). (**e**) Background-subtracted gold particle densities for CB_1 and Cav2.2 of P-face fragments attached to somatic E-face membranes are shown. (**f-h**) Perisomatic axon terminals contain either Cav2.2 (t2 in **g**) or Cav2.1 (t1 in **g**) subunit, while an excitatory axon terminal contains both subunits (orange, in **h**). Enlarged views of boxed areas on **g** are shown on the bottom and on the right. (**i**) Background-subtracted gold particle densities for Cav2.2 and Cav2.1 subunits measured in axon terminal fragments attached to E-face somatic membranes. In panels **a, c, d, f, g, h** putative AZs are

colored for illustrative purposes. In **b, e, i** every symbol represents a P-face fragment with an area between 0.010 μm^2 and 0.207 μm^2 (n= 1 rat). str. pyr.: stratum pyramidale. gp: guinea pig, rb: rabbit, d: dendrite. All scale bars: 200 nm.

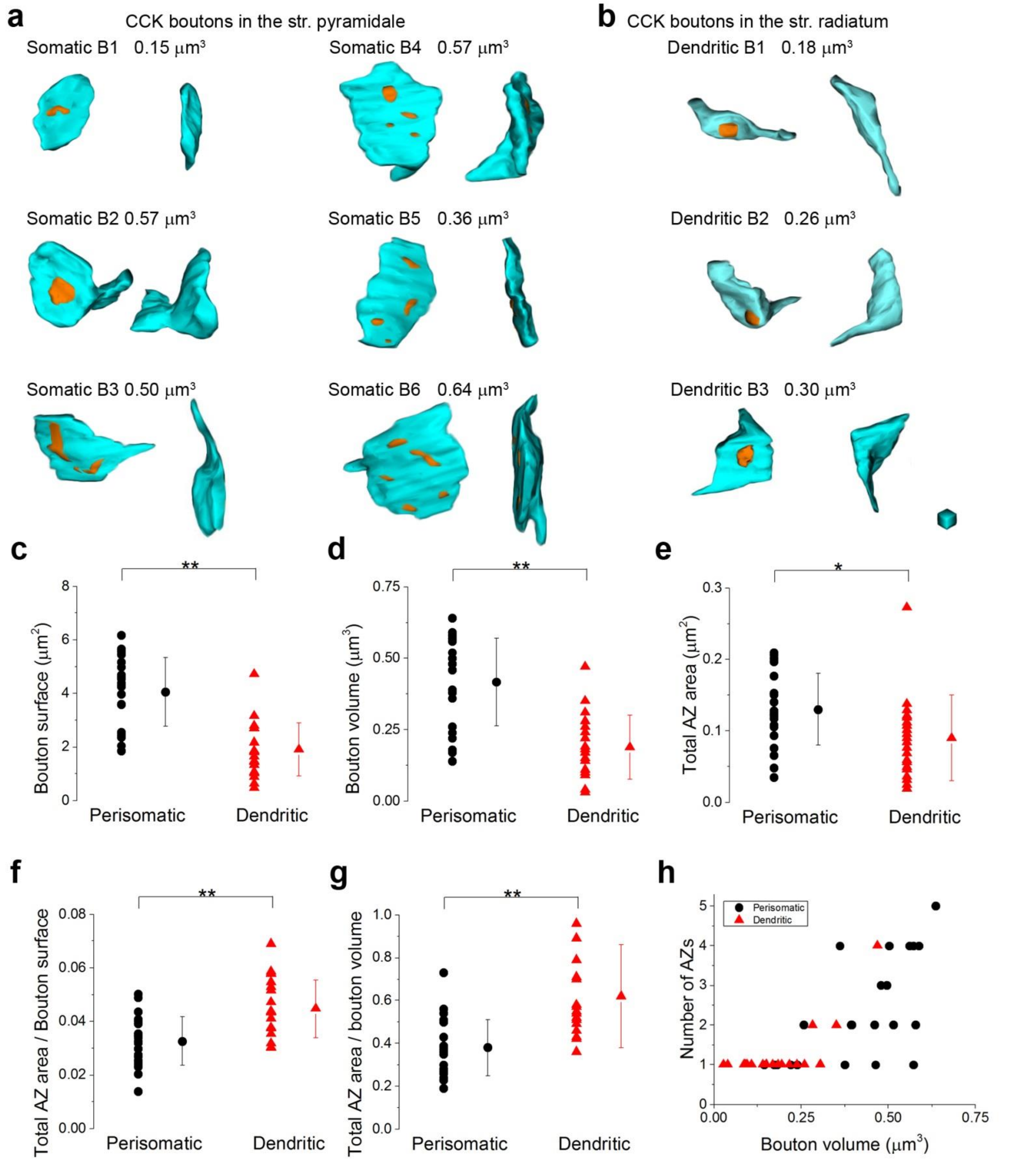

Supplementary Figure 4. Three dimensional reconstructions of cholecystochinin-immunopositive boutons from serial ultrathin sections in the strata pyramidale and radiatum. (**a, b**) Boutons innervating somata (**a**) and dendrites (**b**) show large variability in their volumes, active zone (AZ) number and AZ area. (**c**-**h**) The bouton surface (**c**), the bouton volume (**d**) and the total AZ area (**e**) of the soma-targeting boutons are significantly larger than those of the dendritic ones. The total AZ areas normalized to the bouton surface (**f**) or to the bouton volume (**g**) are significantly larger in the stratum radiatum compared to those in the stratum pyramidale. (**h**) Soma-targeting boutons possess more AZs than dendritic axon terminals ($p = 0.002$). Scale: **a-b**, side of the scale cube, 250 nm; volume of the scale cube, 0.016 μm^3. Unpaired t-test: * $p = 0.01$, ** $p < 0.001$.

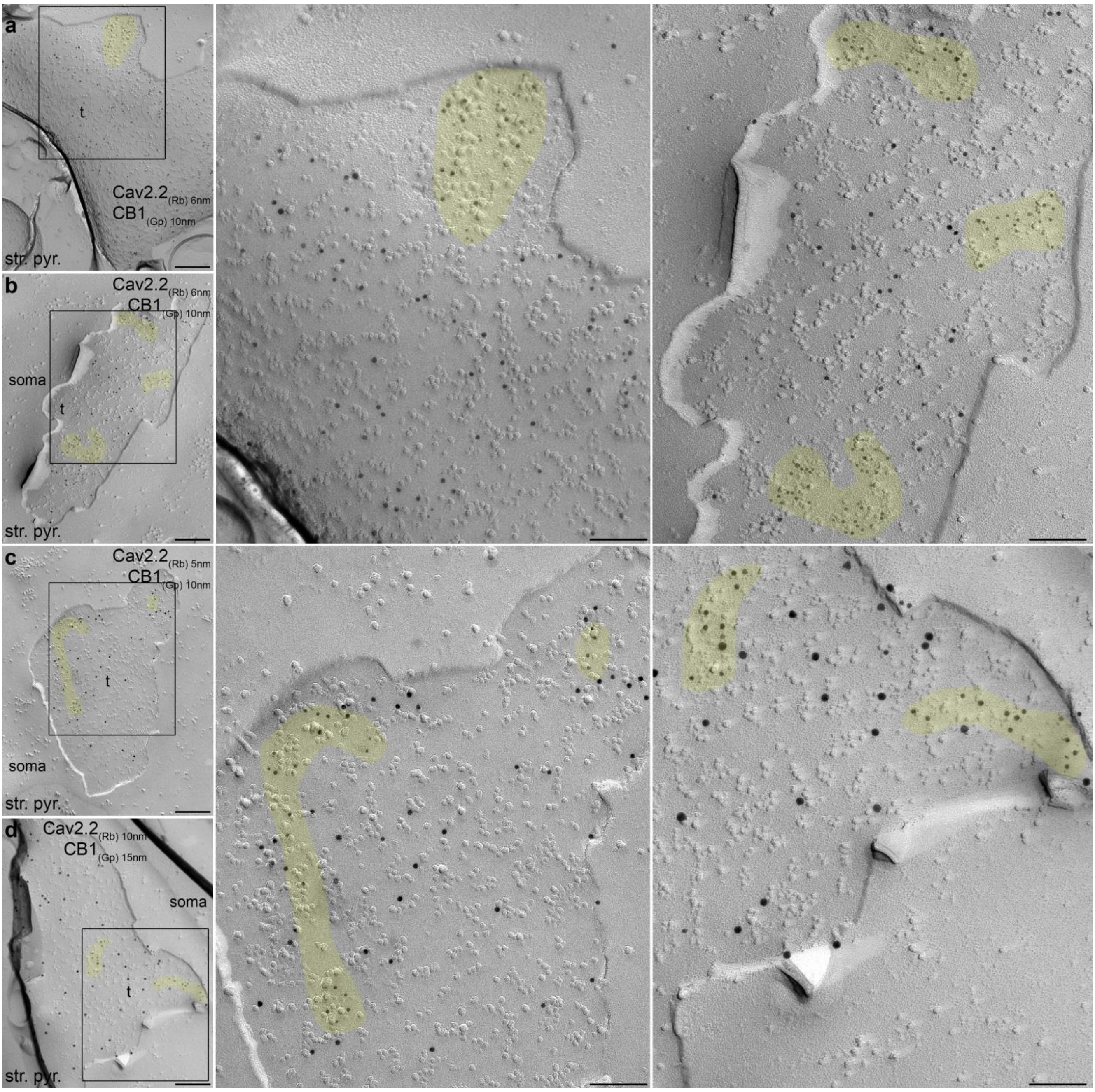

Supplementary Figure 5. Distribution of Cav2.2 subunit on perisomatic CB_1-immunoreactive axon terminals (t) in the mouse hippocampal CA3 area. Double labeling experiments were performed against CB_1 and Cav2.2 subunit and visualized with IgGs coupled to gold particles with differing size: Cav2.2 subunit with 6 nm (**a-b**), 5 nm (**c**), 10 nm (**d**) gold particles; CB_1 with 10 nm (**a-c**) and 15 nm (**d**) gold particles. Similar results were obtained with many different combinations of different secondary antibodies. Enlarged views of boxed areas are shown on the right. Note the various shapes of Cav2.2 populated regions (yellow). str. pyr.: stratum pyramidale, gp: guinea pig, rb: rabbit. Scale bars: **a-d**, 200 nm, enlarged views, 100 nm.

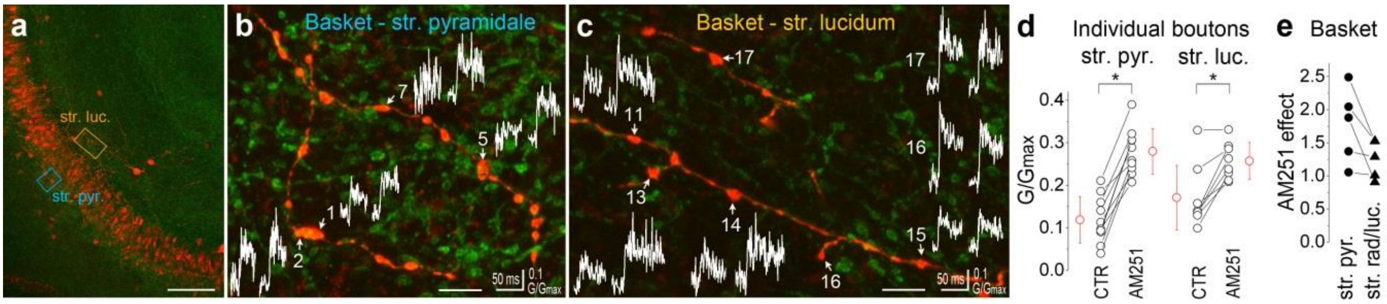

Supplementary Figure 6. [Ca^{2+}] transients in individual axon terminals of a single basket cell in different layers exhibit different tonic CB_1-mediated inhibition. (**a–c**) Confocal maximum intensity projection Z stack images (**a**, low magnifications, **b-c** higher magnifications) of an immunolabeled slice for CB_1 (green) and biocytin (red). Note that cholecystochinin-positive interneurons and pyramidal cells are also red due to their DsRed content (**a**). Bouton strings of a basket cell were imaged in the framed areas and are shown at higher magnifications in (**b**) and (**c**). Action potential-evoked [Ca^{2+}] transients are shown in control conditions (left traces) and in the presence of 100 nM AM251 (right traces) for individual boutons. Note the variability of the [Ca^{2+}] transients in control conditions within a bouton string. (**d**) The effect of AM251 on the amplitude of [Ca^{2+}] transients of individual boutons (paired t-test, $p < 0.001$, $n = 10$ for str. pyramidale and $p = 0.002$, $n = 8$ for stratum lucidum). (**e**) The mean AM251 effects seem to be larger in stratum pyramidale than in stratum lucidum in basket cells, but the effect did not reach significance (paired t-test, $p = 0.056$).

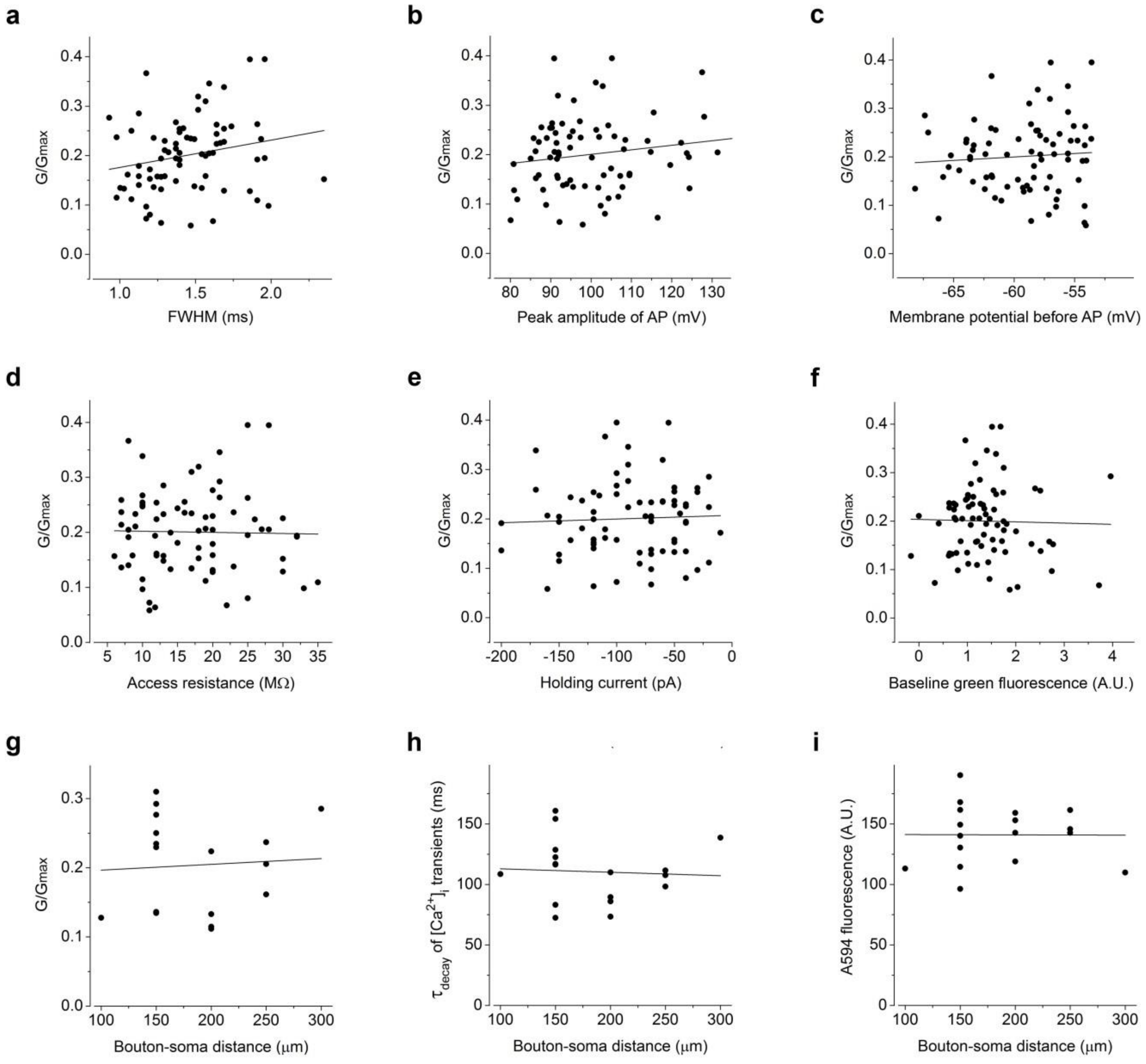

Supplementary Figure 7. [Ca^{2+}] transients are independent of bouton-soma distance and electrophysiological properties of cells. **(a-f)** The amplitudes of [Ca^{2+}] transients do not depend on the width of the somatic action potential (AP) (**a**, r = 0.21, p = 0.066, n = 76), the peak amplitude of the AP (**b**, ρ = 0.099, p = 0.40, n = 76), the membrane potential before the AP (**c**, ρ = 0.066, p = 0.57, n = 76), the access resistance (**d**, ρ = -0.045, p = 0.70, n = 76), the holding current (**e**, ρ = 0.059, p = 0.61, n = 76) or the "baseline" Fluo-5F fluorescence before the AP (**f**, ρ = -0.0043, p = 0.97, n = 75) measured after 60 minutes of dye-loading. (**g-i**) The distance between the recorded bouton string and the soma (axon was traced back and the length was measured in 3D) does not affect the peak of [Ca^{2+}] transients (**g**, ρ = -0.063, p = 0.81, n = 17), the decay of transients (**h**, ρ = -0.16, p = 0.54, n = 17) and the Alexa-594 signal (**i**, ρ = 0.031, p = 0.90, n = 17) after an hour of dye-loading period. Symbols represent individual cells. Datasets were tested by Shapiro-Wilk normality test. If the dataset did not differ from a normal distribution and the sample size was larger than 30, then Pearson's correlation was used (only panel **a**, indicated with r value), otherwise Spearman's rank-order correlation was performed (ρ values).

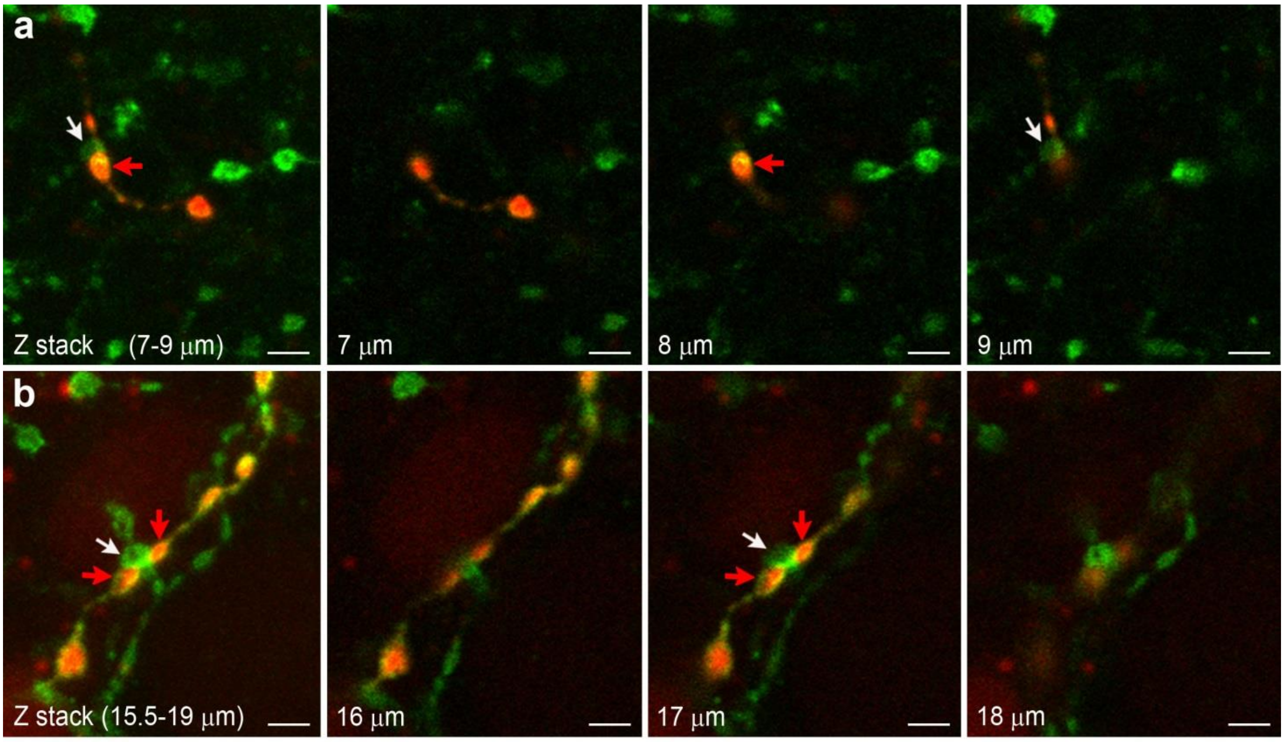

Supplementary Figure 8. Sparse CB_1 immunolabeling allows the measurement of the total CB_1 content of individual boutons. (**a-b**) Biocytin-filled boutons (red arrows) seem to overlap with biocytin unlabeled boutons (white arrows) on the maximum intensity projection Z stack images (1st panels) apparently precluding the measurements of their CB_1 immunoreactivity. However, the overlapping boutons could be in either different (**a**) or identical (**b**) focal planes. In the former, the CB_1 immunoreactivity of the bouton could be reliably measured in a single confocal section (3rd panel in **a**) and was included in the study. When a biocytin unlabeled bouton (white arrow in **b**) was in close proximity to labeled boutons (red arrows in **b**) within a focal plane (3rd panel in **b**), the boutons were discarded from the analysis.
